# Sarcopenia Is a Prognostic Factor of Adverse Effects and Mortality in Patients With Tumour: A Systematic Review and Meta‐Analysis

**DOI:** 10.1002/jcsm.13629

**Published:** 2024-11-11

**Authors:** Yujie Zhang, Jingjing Zhang, Yunfan Zhan, Zhe Pan, Qiaohong Liu, Wei'an Yuan

**Affiliations:** ^1^ Clinical Research Center Shuguang Hospital Affiliated to Shanghai University of Traditional Chinese Medicine Shanghai China

**Keywords:** chemoradiotherapy, meta‐analysis, prognosis, sarcopenia, tumour

## Abstract

**Background:**

The relationship between sarcopenia and the prognosis of patients with tumours who received radio‐ and/or chemotherapy still needs to be determined. In this study, we aim to investigate the relationship between sarcopenia and adverse effects and mortality in patients with tumours that received radio‐ and/or chemotherapy, stratified by study design, tumour category, the method sarcopenia assessed, treatment options, study location and among other factors.

**Methods:**

PubMed, Web of Science and Embase were searched from inception to 15 August 2024, without language restrictions and with a manual search of references for additional articles retrieval. Cohort studies of ≥ 100 patients with tumours that evaluated the association between sarcopenia or muscle mass and the adverse effects or overall survival induced by radio‐ and/or chemotherapy were included.

**Results:**

Thirty‐nine studies were included, involving 8966 patients with tumours, including 3383 patients with sarcopenia. The pooled prevalence of sarcopenia in patients with tumours was 0.42 (95% CI 0.36–0.48, *p* < 0.001) overall. The prevalence of sarcopenia is higher in Oceania patients 0.60 (95% CI 0.28–0.89, *p* < 0.001), those with reproductive tumour 0.57 (95% CI 0.30–0.83, *p* < 0.001), and sarcopenia assessed by the lumbar‐skeletal muscle index 0.46 (95% CI 0.39–0.53, *p* < 0.001) than in other subgroups, but not show significant differences in sex. Sarcopenia was associated with an increased risk of adverse effects in patients who received radio‐ and/or chemotherapy, with a relative risk (RR) of 1.44 (95% CI 1.21–1.71, *p* < 0.001). Retrospective studies (RR = 1.49; 95% CI 1.24–1.79; *p* < 0.001), sarcopenia assessed by other methods (RR = 2.98; 95% CI 1.52–5.87; *p* < 0.001), and patients in Europe (RR = 1.92; 95% CI 1.15–3.22; *p* = 0.013), received chemoradiotherapy (RR = 1.47; 95% CI 1.23–1.76; *p* < 0.001), and with head and neck tumours (RR = 1.54; 95% CI 1.17–2.01; *p* = 0.010) had higher relative risk than other subgroups. Sarcopenia was also associated with reduced overall survival in patients with tumours, with a pooled hazard ratio (HR) of 1.66 (95% CI 1.40–1.96, *p* < 0.001). Prospective studies (HR = 1.72; 95% CI 0.97–3.07; *p* = 0.065), sarcopenia assessed by the cervical‐skeletal muscle index (HR = 2.66; 95% CI 1.73–4.09; *p <* 0.001), and patients in Asia (HR = 1.91; 95% CI 1.50–2.42; *p* < 0.001), received chemoradiotherapy (HR = 1.85; 95% CI 1.46–2.45; *p* < 0.001) and with head and neck tumours (HR = 2.35; 95% CI 1.88–2.95; *p* < 0.001) had higher HR than other subgroups.

**Conclusions:**

Sarcopenia was associated with a higher risk of adverse effects and mortality in patients with tumours received radio‐ and/or chemotherapy.

## 1 Introduction

Sarcopenia is defined as a progressive and generalized skeletal muscle disorder, which increased the risk of adverse effects such as falls, fractures, disability and mortality [1, 2, 3, 4], also recognized as a separate disease entity with the issuance of an ICD‐10‐CM code [5]. It was reported in 2022 that a meta‐analysis of sarcopenia incidence at the global and regional levels was carried out, which gave an estimate of the prevalence of severe sarcopenia and sarcopenia according to sociodemographic characteristics worldwide, estimated that the incidence rates of sarcopenia ranged from 10% and 27% [6, 7, 8, 9, 10]. Aging leads to a reduction of skeletal muscle function in terms of muscle mass, strength and muscular endurance [11]. Thus, sarcopenia is a growing concern for aging societies due to its association with a range of adverse outcomes and increased healthcare costs [12, 13].

It is widely believed that tumour is a major cause of secondary sarcopenia [14]. Sarcopenia, on the other hand, has also been found to negatively impact tumour patients' prognoses. According to recent studies, cachexia and sarcopenia, which were previously confused, represent different aspects of muscle wasting [15, 16]. It is estimated that 15% to 50% of tumour patients with weight loss are sarcopenic rather than cachectic [17]. The prognostic value of sarcopenia in various cancers has been assessed in several meta‐analyses, such as resectable and unresectable oesophageal cancer [18, 19], oesophageal cancer [20], ovarian cancer [21] and other types of cancer. However, most have investigated cancer at a particular site in the body. Furthermore, none of the studies address cancer therapy of radio‐ and/or chemotherapy. Radio‐ and/or chemotherapy, as cornerstones in cancer treatment, are commonly accompanied by side effects, including anaemia, thrombocytopenia, appetite loss and diarrhoea, impacting the patient's overall health and treatment outcome. Amino acids and proteins are indispensable components of the body's nutrition, with muscles serving as the body's largest protein reservoir [22, 23]. Muscle loss not only has negative effects on the quality of patients' lives but also reduces efficacy and increases toxicity of anticancer radio‐ and/or chemotherapy and is a risk factor for poor prognosis in patients with tumour [24]. According to the FDA, cancer drug trials are effective if they prolong the survival of cancer patients and improve their clinical symptoms, of which overall survival (OS) is the gold standard [25].

Considering the current high prevalence of tumour and sarcopenia as a common co‐morbid factor of tumours, we believe that it is of great clinical significance to study the epidemiology of sarcopenia in patients with tumours and its role as a risk factor for poor prognosis after radio‐ and/or chemotherapy. Hence, we conducted a systematic review and meta‐analysis to assess sarcopenia (vs. non‐sarcopenia) patients with tumours received radio‐ and/or chemotherapy have a higher risk of adverse effects (AE) and overall survival (OS). Detailed subgroup analysis was conducted to determine the distribution of sarcopenia and risk factor of sarcopenia in AE and OS, stratified by study design, tumour category, the way sarcopenia assessed, treatment options, study location and among other factors.

## 2 Materials and Methods

### 2.1 Search Strategy and Selection Criteria

This meta‐analysis was conducted according to the updated PRISMA [26] (2020) and MOOSE [27] guidelines, and it has been registered in PROSPERO (CRD42024494521).

PubMed, Web of Science and Embase were searched from inception to 15 August 2024, without language restrictions and with a manual search of references for additional articles to retrieve relevant literature reporting the association between sarcopenia and adverse effects or overall survival in patients with tumour received radio‐ and/or chemotherapy.

Search keywords included sarcopenia, tumour, chemotherapy or/and radiotherapy, adverse effects, overall survival and study type. A detailed search strategy can be found in Table S1. We restricted the search to clinical studies and the sample size to no less than 100 cases. In the case of studies with overlapping cohorts, studies with recent data, larger sample sizes or more data available for subgroup analysis were included. Reports of duplicate studies are excluded by checking author lists, parent institutions, sample sizes and results. J.Z. and Y.Z independently screened title and abstract eligibility, based on a pre‐planned list of inclusion and exclusion criteria (Appendix 1), with disputed studies determined by a third Y.Z. in consultation.

### 2.2 Data Extraction and Quality Assessment

A standardized extraction form was used to extract data from selected studies, including the first author, the year of publication, study design, source of study location/study period, definition of sarcopenia, methods of measuring muscle mass or strength, sample size, demographic and diagnosis information (age, sex, diagnosis and treatment options), and study outcomes. If relevant information is missing or unavailable, the author will be contacted for more data and/or clarification.

The Newcastle‐Ottawa Scale was used to assess the quality of included studies (Appendix 2) [28]. Two researchers (Y.Z. and J.Z.) will independently rate the quality of the study, and a third reviewer (Y.Z.) will resolve differences through consensus or discussion.

### 2.3 Statistical Analysis

Statistical analyses were performed using Stata16.0 and R version 4.1.0. The primary outcome was the prevalence of sarcopenia in patients with tumour, AE and OS. The prevalence of sarcopenia was pooled using a meta‐analysis of single proportions, presented as a decimal. Subgroup data were provided according to tumour category, the way sarcopenia assessed, treatment options, study location and among other factors.

The effect of sarcopenia on AE was evaluated by RR and 95% CIs, and the pooled HR and 95% CIs on OS, respectively, using random effects model. Heterogeneity across studies was assessed using the *I* ^2^ statistic and Cochran's *Q* statistic. High heterogeneity was defined as a *p* value of ≤ 0.1 for the *Q* statistic or an *I* ^2^ value of ≥ 50% [29]. Subgroup analyses based on study design, tumour category, the way sarcopenia assessed, treatment options, study location, and meta‐regression based on the sample size, average age, proportion of males, study location, tumour category, treatment options, and the method sarcopenia were used to investigate potential sources of heterogeneity.

To ensure the robustness and reliability of the meta‐analysis findings, sensitivity analysis was conducted using the leave‐one‐out method. Additionally, publication bias was assessed through funnel plot analysis and Egger's and Begg's tests [30].

## 3 Results

### 3.1 Study Retrieval, Characteristics of Included Studies and Prevalence of Sarcopenia

**FIGURE 1:**
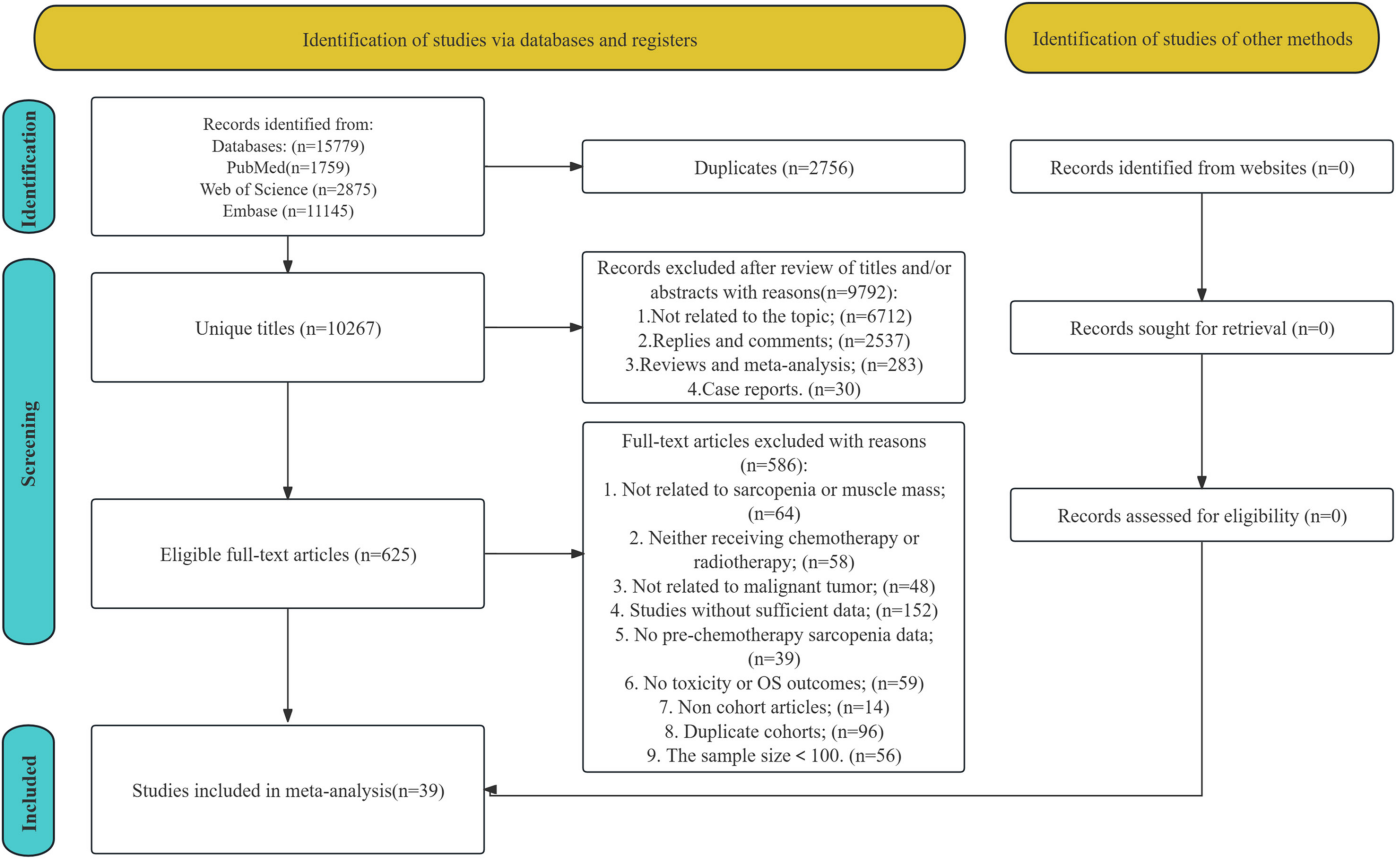
Flowchart of the systematic search and selection process.

Of the 15 779 records identified from the initial search, 2756 duplicates and 9792 irrelevant titles/abstracts were excluded. Out of the remaining 625 articles subjected to full‐text review, this study included 39 cohort studies containing data on 8966 patients (Figure 1) [31, 32, 33, 34, 35, 36, 37, 38, 39, 40, 41, 42, 43, 44, 45, 46, 47, 48, 49, 50, 51, 52, 53, 54, 55, 56, 57, 58, 59, 60, 61, 62, 63, 64, 65, 66, 67, 68, 69]. Table 1 summarizes the characteristics of all studies included in this meta‐analysis. Overall, 23 [31, 36, 37, 41, 42, 43, 44, 45, 46, 47, 48, 49, 50, 51, 52, 53, 60, 62, 64, 66, 67, 68, 69] of 39 included studies were from Asia, 11 [35, 38, 39, 55, 56, 57, 58, 59, 61, 63, 65] studies were from Europe, 3 [34, 40, 54] studies were from Americas, and 2 [32, 33] were from Oceania. Thirty‐five [31, 34, 36, 37, 38, 40, 41, 42, 43, 44, 45, 46, 47, 48, 49, 50, 51, 52, 53, 54, 55, 56, 57, 58, 59, 60, 61, 62, 63, 64, 65, 66, 67, 68, 69] of the 39 studies were retrospective cohort studies, and the other 4 [32, 33, 35, 39] studies were prospective cohort studies. The sample size of the included studies ranged from 101 to 862. The mean age of patients ranged from 45 to 72 years among the included studies. All studies were assessed for NOS and rated as high quality with score = 9 for 32 [31, 32, 33, 35, 36, 37, 38, 39, 40, 41, 42, 43, 44, 46, 47, 48, 49, 51, 52, 54, 55, 56, 57, 58, 59, 61, 62, 64, 66, 67, 68, 69] studies and with score = 8 for the other 7 [34, 45, 50, 53, 60, 63, 65] studies (Table S2).

**TABLE 1:** Summary characteristics of included studies.

Author (year)	Study design	Source of study population/study period	Diagnosis	Treatment	Sample size	Age mean (SD/range) (years)	Sex (male/female)	Methods for measuring muscle mass or strength	Definition of sarcopenia	Outcomes
Abe (2022)	Retrospective study	Japan/2003–2016	Lower digestive tract cancer	Chemoradiotherapy	234	64.5 (57–71)	151/83	CT: L3‐PMI	≤ 5.32 cm^2^/m^2^ for men; ≤ 4.11 cm^2^/m^2^ for women	AE
Ahern (2023)	Prospective study	Australia/NA	Head and neck squamous cell carcinoma	Chemoradiotherapy or radiotherapy	101	60.5 (10.1)	92/9	CT: L3‐SMI	≤ 43 cm^2^/m^2^ for men; ≤ 41 cm^2^/m^2^ for women	OS
Bedrikovetski (2023)	Prospective study	Australia/2019–2022	Advanced rectal cancer	Chemotherapy	118	59.5 (14.3)	74/44	CT: L3‐TPAI	≤ 54.5 cm^2^/m^2^ for men; ≤ 38.5 cm^2^/m^2^ for women	AE
Bruno (2021)	Retrospective study	Brazil/2008–2017	Ovarian adenocarcinoma	Chemotherapy	239	56.3 (11.4)	0/239	CT: L3‐SMI	≤ 38.9 cm^2^/m^2^ for women	AE OS
Charette (2019)	Prospective study	Belgium/2011, 2013–2014	Colorectal cancer	Chemotherapy	217	63 (11)	123/94	CT: L3‐SMI	SMI ≤ 43 cm^2^/m^2^ (BMI < 25 kg/m^2^) and SMI ≤ 53 cm^2^/m^2^ (BMI ≥ 25 kg/m^2^) for male; SMI ≤ 41 cm^2^/m^2^ (for female	OS
Cho (2018)	Retrospective study	South Korea/2006–2015	Head and neck cancer	Chemoradiotherapy	221	59 (18–94)	180/41	CT: L3‐SMI	≤ 49 cm^2^/m^2^ for men; ≤ 31 cm^2^/m^2^ for women	AE OS
Choi (2018)	Retrospective study	Korea/2009–2013	Rectal cancer	Chemoradiotherapy	188	61.3 (27–84)	117/71	CT: L3‐SMI	≤ 52.4 cm^2^/m^2^ for men; ≤ 38.5 cm^2^/m^2^ for women	OS
Chiloiro (2024)	Retrospective study	Italy/2010–2020	Rectal cancer	Radiotherapy	628	63.4 (11.4)	371/257	CT: L3‐SMI	≤ 52.4 cm^2^/m^2^ for men; ≤ 38.5 cm^2^/m^2^ for women	OS
Daly (2018)	Prospective study	Ireland/2012–2016	Foregut cancer	Chemotherapy	225	65.6 (35.8–83.4)	150/75	CT: L3‐SMI	SMI ≤ 43 cm^2^/m^2^ (BMI < 25 kg/m^2^) and SMI ≤ 53 cm^2^/m^2^ (BMI ≥ 25 kg/m^2^) for male; SMI ≤ 41 cm^2^/m^2^ for female	OS
Ganju (2019)	Retrospective study	United States/2012–2016	Head and neck cancer	Chemoradiotherapy	246	—	199/47	CT: L3‐SMI	SMI < 41 cm^2^/m^2^ for female; SMI < 43 cm^2^/m^2^ (BMI < 25) and < 53 cm^2^/m^2^ (BMI > 25) for male	AE OS
Go (2016)	Retrospective study	Korea/2003–2015	Large B‐cell lymphoma	Chemotherapy	187	Sarcopenic 66.5 (24–89) Non‐sarcopenic 60 (17–86)	112/75	CT: T4‐SMI	≤ 44 cm^2^/m^2^ for male; ≤ 31 cm^2^/m^2^ in female	AE
Hua (2020)	Retrospective study	China/2010–2014	Nasopharyngeal carcinoma	Chemoradiotherapy	862	45.84 (10.78)	640/222	CT: C3‐SMI	≤ 18.82 cm^2^/m^2^	AE OS
Huang (2019)	Retrospective study	China/2015–2017	Nasopharyngeal carcinoma	Chemoradiotherapy	394	< 60 35 (88) ≥ 60 44 (11.2)	298/96	CT: L3‐SMI	≤ 40.8 cm^2^/m^2^ for male; ≤ 34.9 cm^2^/m^2^ for female	OS
Huang (2020)	Retrospective study	China/2001–2014	Oesophageal cancer	Chemotherapy	107	54.1 (7.5)	101/6	CT: L3‐SMI	< 52.4 cm^2^/m^2^ for male;< 38.5 cm^2^/m^2^ for female	AE
Huang (2021)	Retrospective study	China/2011–2018	Oral cavity squamous cell	Chemoradiotherapy	175	53.9 (9.9)	151/24	CT: C3‐SMI	≤ 46.7 cm^2^/m^2^ for male; ≤ 30.3 cm^2^/m^2^ for female;	AE OS
Ishizaki (2023)	Retrospective study	Japan/2015–2020	Pancreatic ductal adenocarcinoma	Chemotherapy	180	—	119/61	CT: L3‐SMI	≤ 42 cm^2^/m^2^ for male; ≤ 38 cm^2^/m^2^ for female	OS
Jin (2022)	Retrospective study	China/2013–2017	Pancreatic cancer	Chemotherapy	119	60.2 (8.4)	59/60	CT: L3‐SMI	≤ 41 cm^2^/m^2^ for male; ≤ 38.5 cm^2^/m^2^ for female	OS
Jang (2024)	Retrospective study	Korea/2025–2021	Oesophageal cancer	Chemoradiotherapy	345	63 (36–83)	—	CT: L3‐SMI	SMI < 43 cm^2^/m^2^ (BMI < 25) and < 53 cm^2^/m^2^ (BMI > 25)	OS
Kasahara (2024)	Retrospective study	Japan/2012–2016	Head and neck cancer	Chemoradiotherapy	100	68 ± 9	94/9	CT: L3‐SMI	< 43.2 cm^2^/m^2^	OS
Lee (2018)	Retrospective study	China/2004–2015	Cervical cancer	Chemoradiotherapy	245	63.0 ± 12.7	0/245	CT: L3‐SMI	< 41 cm^2^/m^2^	AE
Lee (2021)	Retrospective study	China/2010–2015	Oral cavity cancer	Chemoradiotherapy	174	51 (45–58)	159/19	CT: L3‐SMI	≤ 52.4 cm^2^/m^2^ for male; ≤ 36.2 cm^2^/m^2^ for female	AE OS
Li (2023)	Retrospective study	China/2011–2015	Metastatic castration‐resistant prostate cancer	Chemotherapy	105	69 (61–75)	105/0	CT: L3‐SMI	≤ 43.65 cm^2^/m^2^	OS
Liu (2024)	Retrospective study	China/2016–2019	Nasopharyngeal carcinoma	Chemoradiotherapy	545	46 (18–69)	396/149	CT: C3‐SMI	≤ 51 cm^2^/m^2^ for male; ≤ 38 cm^2^/m^2^ for female	AE OS
Ma (2021)	Retrospective study	Canada/2010–2016	Gastric and oesophageal cancer	Chemotherapy	175	63 (29–87)	121/54	CT: L3‐SMI	≤ 45.4 cm^2^/m^2^ for male; ≤ 34.4 cm^2^/m^2^ for female	OS
Martin (2020)	Retrospective study	Germany/2007–2018	Anal squamous cell carcinoma	Chemoradiotherapy	114	58.5 (26–87)	58/56	CT: L3‐SMI	≤ 41.3 cm^2^/m^2^ for male; ≤ 34.2 cm^2^/m^2^ for female	AE OS
Martin (2022)	Retrospective study	France/2015–2018	Digestive cancer	Chemotherapy	244	69.0 (59–74)	135/109	HGS	< 27 kg for male; < 16 kg for female	AE
McSweeney (2023)	Retrospective study	UK/2017–2020	Oesophageal cancer	Chemoradiotherapy	135	72 (66–76)	52/83	CT: T12‐SMI	≤ 24.1 cm^2^/m^2^ for male; ≤ 18.3 cm^2^/m^2^ for female	OS
Nilsson (2021)	Retrospective study	Sweden/2009–2017	Anal cancer	Chemoradiotherapy	106	63.8 (44–82)	22/84	CT: L3‐SMI	≤ 41.3 cm^2^/m^2^ for male; ≤ 34.2 cm^2^/m^2^ for female	AE
Pielkenrood (2020)	Prospective study	The Netherlands/2013–2016	Spinal metastases	Radiotherapy	310	67 (60–75)	194/116	CT: L3‐SMI	≤ 52.4 cm^2^/m^2^ for male; ≤ 38.5 cm^2^/m^2^ for female	OS
Qian (2022)	Retrospective study	China/2013–2017	Oesophageal squamous cell cancer	Radiotherapy or chemoradiotherapy	213	—	160/53	CT: L3‐SMI	≤ 53 cm^2^/m^2^ for male; ≤ 41 cm^2^/m^2^ for female	OS
Stangl‐Kremser (2020)	Retrospective study	Austria/2005–2015	Castration‐resistant prostate cancer	Chemotherapy	186	68.8 (64.6–75.0)	186/0	CT: L3‐SMI	≤ 55 cm^2^/m^2^ for male	OS
Takeda (2018)	Retrospective study	Japan/2014–2011	Rectal cancer	Chemoradiotherapy	144	61.2 (32–81)	102/42	CT: L3‐SMI	≤ 45 cm^2^/m^2^ for male; ≤ 33.8 cm^2^/m^2^ for female	OS
Thureau (2021)	Prospective study	France/2014–2018	Head and neck cancer	Radiotherapy or radiochemotherapy	243	61.13 (9.04)	187/56	CT: L3‐SMI	≤ 52.4 cm^2^/m^2^ for male; ≤ 38.5 cm^2^/m^2^ for female	OS
Watanabe (2023)	Retrospective study	Japan/2010–2020	Non‐small cell lung cancer	Chemoradiotherapy	106	63 (21–76)	20/86	CT: T12‐SMI	≤ 12.4 cm^2^/m^2^ for male; ≤ 10.69 cm^2^/m^2^ for female	AE OS
Willemsen (2020)	Retrospective study	The Netherlands/2013–2016	Head and neck squamous cell carcinoma	Chemoradiotherapy	137	59 (8)	93/44	BIA	< 17 kg/m^2^ for male; < 15 kg/m^2^ for female	AE
Xu (2021)	Retrospective study	China/2014–2016	Oesophageal squamous cell carcinoma	Chemoradiotherapy	184	62 (54–75)	141/43	CT: L3‐SMI	≤ 47.24 cm^2^/m^2^ for male; ≤ 36.92 cm^2^/m^2^ for female	AE OS
Yamahara (2021)	Retrospective study	Japan/2009–2016	Head and neck cancer	Chemotherapy	164	72 (41–92)	143/21	CT: C3‐SMI	≤ 43.2 cm^2^/m^2^	OS
Yu (2020)	Retrospective study	Korea/2004–2008	Gastric cancer	Chemoradiotherapy	440	56 (28–77)	282/158	CT: L3‐SMI	≤ 49 cm^2^/m^2^ for male; ≤ 31 cm^2^/m^2^ for female	AE OS
Zhang (2021)	Retrospective study	China/2016–2018	Gastric cancer	Chemotherapy	110	61.5 (53–67)	80/30	CT: L3‐SMI	≤ 52.4 cm^2^/m^2^ for male; ≤ 38.5 cm^2^/m^2^ for female	OS

Abbreviations: AE: adverse effects; OS: overall survival.

Prevalence data for sarcopenia were available in 37 [31, 32, 33, 34, 35, 36, 37, 38, 39, 40, 41, 42, 43, 44, 45, 46, 47, 48, 49, 50, 51, 53, 54, 55, 56, 57, 58, 59, 60, 61, 62, 63, 64, 65, 66, 67, 68, 69] of 39 included studies (*n* = 8754), generating a pooled prevalence of 0.42 (95% CI 0.36–0.48, *p* < 0.001) (Figure S1). Subgroup analysis showed that the prevalence of sarcopenia in patients was 0.41 (95% CI 0.31–0.51, *p* < 0.001) with digestive tumours, 0.43 (95% CI 0.34–0.52, *p* < 0.001) with head and neck tumours, 0.57 (95% CI 0.30–0.83, *p* < 0.001) with reproductive tumours, 0.28 (95% CI 0.22–0.35, *p* < 0.001) for other tumours (Figure S2A); was 0.24 (95% CI 0.20–0.28, *p* < 0.001) when sarcopenia defined by third/twelfth thoracic‐skeletal muscle index (T3/12‐SMI), 0.46 (95% CI 0.39–0.53, *p* < 0.001) by third/fourth lumbar‐skeletal muscle index (L3/4‐SMI), 0.30 (95% CI 0.17–0.45, *p* < 0.001) by third cervical‐skeletal muscle index (C3‐SMI), and 0.29 (95% CI 0.09–0.54, *p* = 0.008) by other methods (Figure S2B); was 0.43 (95% CI 0.35–0.51, *p* < 0.001) in patients in Asia, 0.60 (95% CI 0.28–0.89, *p* < 0.001) in patients in Oceania, 0.44 (95% CI 0.30–0.59, *p* < 0.001) in patients in Americas, 0.34 (95% CI 0.24–0.44, *p* < 0.001) in patients in Europe (Figure S2C); but not show significant differences in sex of 0.42 (95% CI 0.32–0.52, *p* < 0.001) in male patients and 0.40 (95% CI 0.31–0.48, *p* < 0.001) in female patients (Figure S2D).

### 3.2 Association Between Sarcopenia and AE

#### 3.2.1 Analysis of the Overall Cohort

**FIGURE 2:**
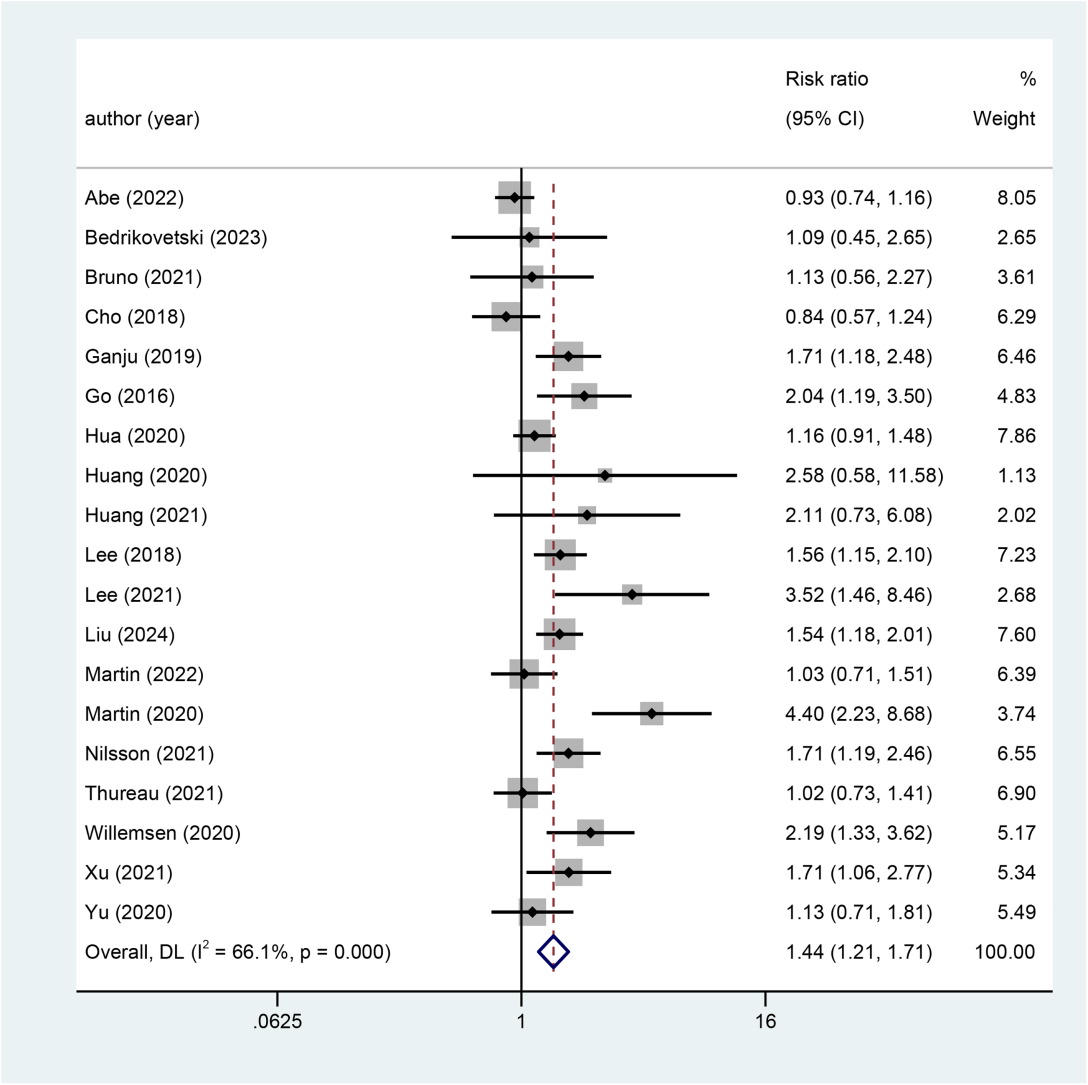
Forest plot for the association between sarcopenia and AE.

From the included 19 studies [31, 33, 34, 36, 40, 41, 42, 44, 45, 50, 51, 53, 55, 56, 58, 63, 65, 66, 68] (*n* = 4903) that provided data on univariate analysis, sarcopenia was associated with an increased risk of AE in patients receiving radio‐ and/or chemotherapy, with RR of 1.44 (95% CI 1.21–1.71, *p* < 0.001) (Figure 2). The heterogeneity between studies was high (*I* ^2^ = 66.1%). The level evidence was low according to GRADE (Table S3).

#### 3.2.2 Sensitivity Analysis

Our sensitivity analyses excluding one study at a time and then pooling the remaining studies showed RRs of toxicity ranging from 1.36 to 1.48, was similar to that the main analysis of 1.44 (95% CI 1.21–1.71), suggesting that our results were robust (Figure S3) (Table S4).

#### 3.2.3 Subgroup Analysis

**FIGURE 3:**
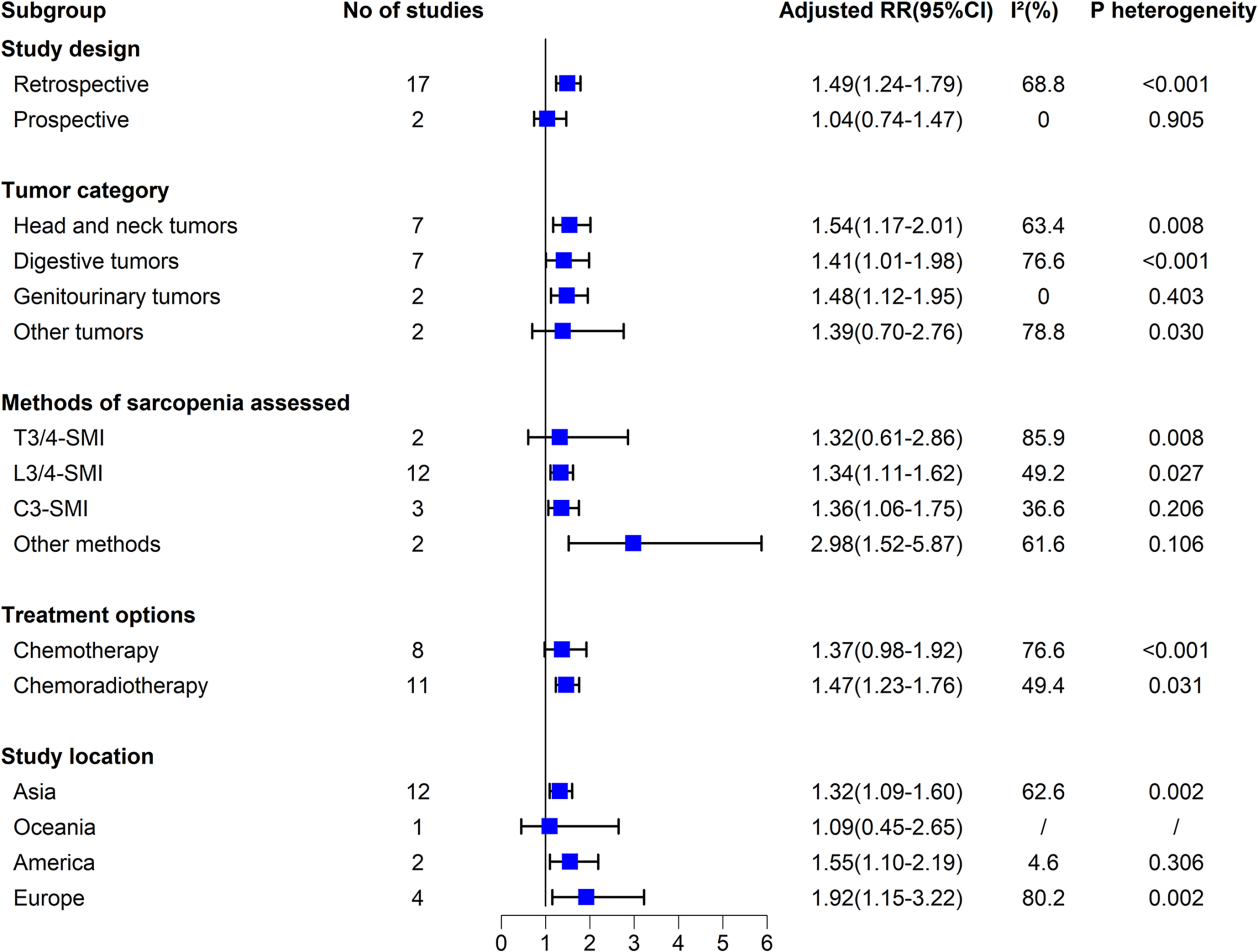
Association of sarcopenia and risk of AE in patients with tumour received radio‐ and/or chemotherapy in study subgroups.

We found that sarcopenia was consistently associated with a higher risk of AE across all subgroups analysed. Specifically, sarcopenia (vs. non‐sarcopenia) was associated with a significantly increased risk of AE in both prospective and retrospective studies with RR of 1.04 (95% CI 0.74–1.47, *p* = 0.818) and 1.49 (95% CI 1.24–1.79, *p* < 0.001), respectively; in patients with digestive tumours of 1.41 (95% CI 1.01–1.98; *p* = 0.043), with genitourinary tumours of 1.48 (95% CI 1.12–1.95; *p* = 0.005), with head and neck tumours of 1.54 (95% CI 1.17–2.01; *p* = 0.010) and 1.39 (95% CI 0.70–2.76; *p* = 0.341) with other tumours; regardless of the method sarcopenia assessed, with T3/4‐SMI of 1.32 (95% CI 0.61–2.86; *p* = 0.478), with L3/4‐SMI of 1.34 (95% CI 1.11–1.62; *p* = 0.003), with C3‐SMI of 1.36 (95% CI 1.06–1.75; *p* = 0.016) and with other measurement such as BIA (Bioelectric impedance analysis) and HGS (hand grip strength) of 2.98 (95% CI 1.52–5.87; *p* = 0.002); in patients received either chemoradiotherapy or chemotherapy with RR of 1.47 (95% CI 1.23–1.76; *p* < 0.001) and 1.37 (95% CI 0.98–1.92; *p* = 0.063), respectively; and patients in different regions of which 1.32 (95% CI 1.09–1.60; *p* = 0.004) in Asia, 1.92 (95% CI 1.15–3.22; *p* = 0.013) in Europe, 1.55 (95% CI 1.10–2.19; *p* = 0.012) in America, and 1.09 (95% CI 0.45–2.65; *p* = 0.841) in Oceania (Figure 3).

#### 3.2.4 Publication Bias

Funnel pattern was used to detect publication bias of the main outcomes. As shown in Figure S4A, the funnel pattern was asymmetric, indicating that there might have been publication bias. This was further confirmed by Egger's (*p* = 0.026 < 0.05) (Figure S4B) and Begg's tests (*p* = 0.041 < 0.05) (Figure S4C).

#### 3.2.5 Meta‐Regression Analyses

Meta‐regression analyses indicated no association of RR with the sample size (coefficients 0.9994, 95% CI 0.9978–1.0010; *p* = 0.339), participants' average age (coefficients 1.0460, 95% CI 0.9714–1.1264; *p* = 0.167), proportion of males (coefficients 1.3824, 95% CI 0.6081–3.1427; *p* = 0.335), study location (coefficients 1.0958, 95% CI 0.8189–1.4662; *p* = 0.432), tumour category (coefficients 1.3192, 95% CI 0.9735–1.7875; *p* = 0.065), treatment options (coefficients 1.1669, 95% CI 0.6179–2.2038; *p* = 0.537), and the method sarcopenia assessed (coefficients 1.4256, 95% CI 0.9500–2.1393; *p* = 0.072) (Table S5).

### 3.3 Association Between Sarcopenia and OS

#### 3.3.1 Analysis of the Overall Cohort

**FIGURE 4:**
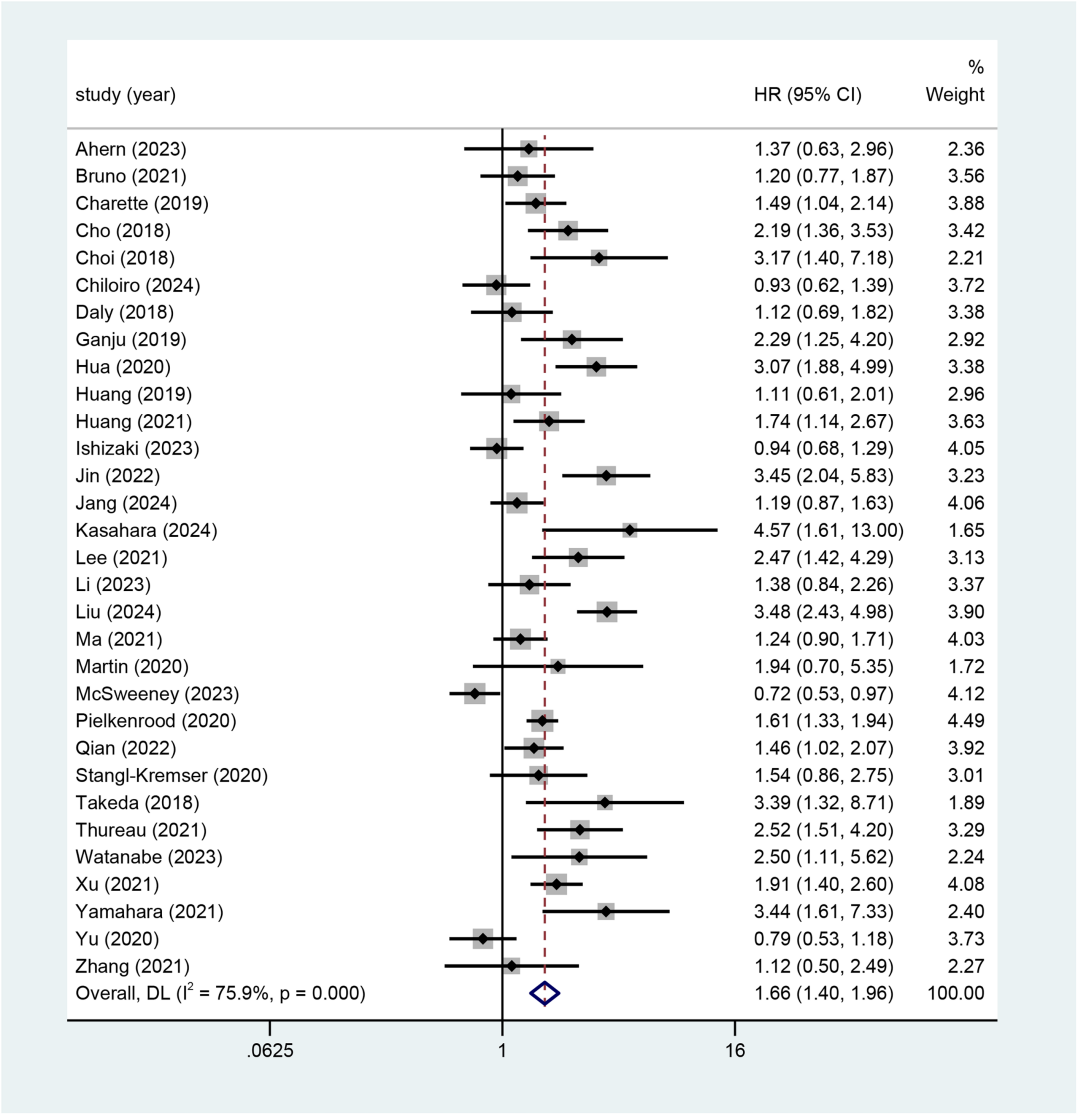
Forest plot for the association between sarcopenia and the OS.

From 31 studies [32, 34, 35, 36, 37, 38, 39, 40, 42, 43, 45, 46, 47, 48, 49, 51, 52, 53, 54, 55, 57, 59, 60, 61, 62, 63, 64, 66, 67, 68, 69] (*n* = 7588) that provided data on univariate analysis, sarcopenia was associated with an increased risk of mortality, with a pooled HR of 1.66 (95% CI 1.40–1.96; *p* < 0.001) (Figure 4). There was high heterogeneity between studies (*I* ^2^ = 75.9%, *p* < 0.001). The level of evidence was low according to the GRADE (Table S6).

#### 3.3.2 Sensitivity Analysis

Our sensitivity analyses, excluding one study at a time and then pooling the remaining studies, showed HRs of OS were similar to that main analysis at 1.66 (95% CI 1.40–1.96), suggesting that our results were robust (Figure S5).

#### 3.3.3 Subgroup Analysis

**FIGURE 5:**
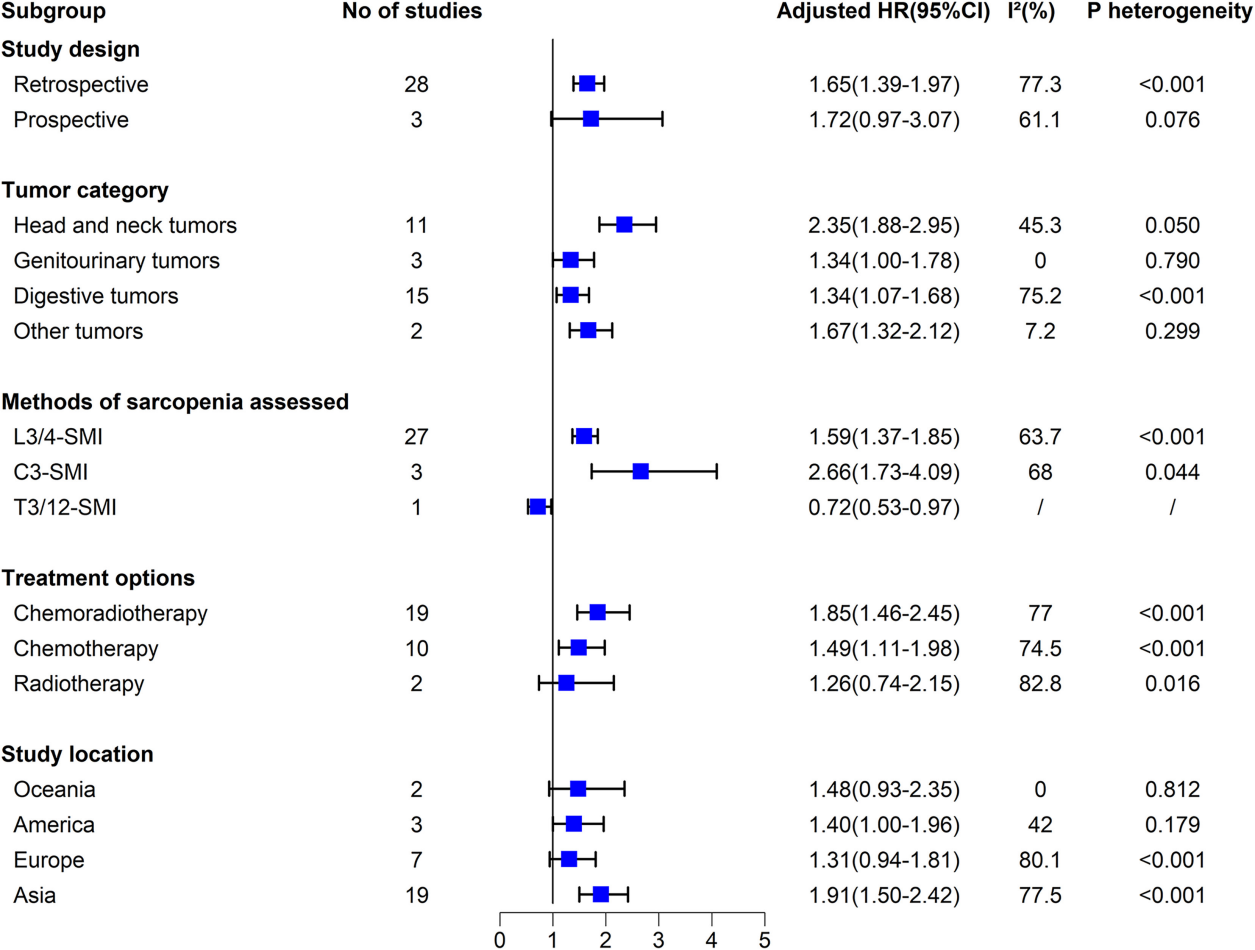
Association of sarcopenia and risk of mortality in patients with tumour received radio‐ and/or chemotherapy in study subgroups.

We found that tumour patients with sarcopenia receiving radio‐ and/or chemotherapy were consistently associated with a higher risk of mortality across all subgroups analysed. Specifically, sarcopenia (vs. non‐sarcopenia) was associated with a significantly increased risk of mortality in both prospective and retrospective studies with HR of 1.72 (95% CI 0.97–3.07; *p* = 0.065) and 1.62 (95% CI 1.39–1.97; *p* < 0.001), respectively; in patients with digestive tumours of 1.34 (95% CI 1.07–1.68; *p* = 0.010), with genitourinary tumours of 1.34 (95% CI 1.00–1.78, *p* = 0.047), with head and neck tumours of 2.35 (95% CI 1.88–2.95, *p* < 0.001), and 1.67 (95% CI 1.32–2.12; *p* < 0.001) with other tumours; regardless of the method sarcopenia assessed, with T3/12‐SMI of 0.72 (95% CI 0.53–0.97; *p* = 0.033), with L3/4‐SMI of 1.59 (95% CI 1.37–1.85; *p* < 0.001), and with C3‐SMI of 2.66 (95% CI 1.73–4.09; *p* < 0.001); in patients received radio‐ and/or chemotherapy with HR of 1.26 (95% CI 0.74–2.15; *p* = 0.395), 1.49 (95% CI 1.11–1.98; *p* = 0.007) and 1.85 (95% CI 1.46–2.45; *p* < 0.001), respectively; and patients in different regions of which 1.91 (95% CI 1.50–2.42; *p* < 0.001) in Asia, 1.31 (95% CI 0.94–1.81; *p* = 0.107) in Europe, 1.40 (95% CI 1.00–1.96; *p* = 0.047) in Americas, and 1.48 (95% CI 0.93–2.35; *p* = 0.100) in Oceania (Figure 5).

#### 3.3.4 Publication Bias

A symmetrical appearance was checked in the funnel plot (Figure S6A). Most of the studies included lay within the 95% CI line. Hidden publication bias was tested using Egger's test (*p* = 0.063) (Figure S6B) and Begg's test (*p* = 0.67) (Figure S6C), which suggested that there was no significant publication bias among the included studies.

#### 3.3.5 Meta‐Regression Analyses

Meta‐regression analyses showed no association of pooled HR with the sample size (coefficients −0.0001, 95% CI −0.0121 to 0.0119; *p* = 0.984), participants' average age (coefficients −0.0199, 95% CI −0.3086 to 0.2689; *p* = 0.887), proportion of males (coefficients 0.1851, 95% CI −7.3203 to 7.6904; *p* = 0.959), study location (coefficients −0.0392, 95% CI −1.3262 to 1.2477; *p* = 0.950), tumour category (coefficients 0.0384, 95% CI −1.6012 to 1.6780; *p* = 0.961), treatment options (coefficients 0.4133, 95% CI −2.5161 to 3.3427; *p* = 0.770) and the method sarcopenia assessed (coefficients 0.3996, 95% CI −3.4716 to 4.2708; *p* = 0.831) (Table S7).

## 4 Discussion

In the systematic review and meta‐analysis of 39 studies involving 3383 tumour patients with sarcopenia who received radio‐ and/or chemotherapy, sarcopenia was associated with a higher risk of AE and mortality. Precisely, those with sarcopenia had a ~1.44 times risk of AE and ~1.66 times risk of mortality higher than those without sarcopenia. The association was further supported by the consistent significance across subgroups based on study design, tumour category, the way sarcopenia assessed, treatment options, study location, and among other factors, which consistently demonstrated the predictive value of sarcopenia in anticipating radio‐ and/or chemotherapy toxicity and mortality. In detail, patients in Europe, patients received chemoradiotherapy, and patients with head and neck tumours had a higher risk of adverse effects than other subgroups. Patients in Asia, patients received chemoradiotherapy, and patients with head and neck tumours had a higher risk of mortality than other subgroups. Considering there was heterogeneity in the results, sensitivity analysis and meta‐regression analyses, in addition to subgroup analyses, were performed to explore the sources of heterogeneity. However, we did not find the source of heterogeneity through these ways; we inferred that the heterogeneity mentioned above might come from the different study settings and populations, not just a single factor. We also reported an overall prevalence of sarcopenia of 42% in tumour patients with sarcopenia, which was higher when sarcopenia was defined by L3/4‐SMI at 46%, higher in patients of Oceania at 60%, and higher in patients with reproductive tumours at 57%, but no significant differences were found between men and women.

Multiple factors are involved in the AE and mortality in patients with tumours and sarcopenia who received radio‐ and/or chemotherapy. Mechanistically, sarcopenia may affect the prognosis of tumour patients in numerous ways. First of all, sarcopenia, as an aging‐related disease, causes physiological changes, such as changes in body composition, metabolic capacity, and receptor function, which have a profound impact on the pharmacokinetics and pharmacodynamics of drugs [70]. Changes in body composition caused by sarcopenia may change the metabolic rate and distribution of drugs, thereby affecting drug efficacy and toxicity [71]. In addition, there is a correlation between the loss of muscle mass and the immune status of cancer patients [72, 73]. Muscles are not only the source of strength but also important immune regulatory organs. Various myokines (such as myogenins) produced by muscles regulate immune responses. The findings of Anoveros‐Barrera [74] et al. found a positive correlation between the number of immune cells and muscle mass status in cancer patients. Research by Nelke et al. confirmed that immune aging is related to changes in myokines produced in large quantities by muscles, such as interleukin‐6 [75]. Patients with sarcopenia may have reduced immune function due to reduced myokine, which plays a key role in anti‐tumour immunity. Finally, sarcopenia may also indirectly affect the effectiveness of treatment by affecting the patient's overall health and tolerance. The loss of muscle mass may cause patients to lose physical fitness and be unable to tolerate standard doses of chemotherapy and radiotherapy, which may require lowering the treatment dose or adjusting the treatment plan. The study by Prado et al. found that many chemotherapy drugs, such as capecitabine, paclitaxel and docetaxel, were less effective in breast cancer patients with sarcopenia [76]. The same conclusion was also confirmed in Shachar's study [77]. It should be noted that radiotherapy and chemotherapy itself can also cause sarcopenia, and the two will form a vicious cycle [78].

There has been no meta‐analysis of sarcopenia as a prognostic factor of AE and mortality in patients with tumours followed by radio‐ and/or chemotherapy. Most of the previous studies focus on the relationship of sarcopenia with a prognosis of a single tumour, such as hepatocellular carcinoma [79, 80], breast cancer [81], gastric cancer [82], and the relationship of sarcopenia with a prognosis of cancer patients who received operative treatment, such as urothelial carcinoma [83], gastrointestinal cancer [84], and oral cavity squamous cell carcinoma [85]. Except for the study of Hans‐Jonas [82], the conclusions among other studies were consistent that sarcopenia is associated with poor prognosis in patients with different tumours. Our study builds upon the foundation laid by previous systematic reviews and meta‐analyses that have explored the role of sarcopenia in individual cancer types. Unlike previous studies, our research takes a broader approach by evaluating the impact of sarcopenia across multiple cancer types, providing a more comprehensive understanding of its prognostic significance. And through rigorous methodology, more reliable conclusions can be drawn about the incidence of sarcopenia in patients with tumour and the relationship between sarcopenia and the adverse effects and prognosis in patients received radio‐ and/or chemotherapy. Because of the close relationship between sarcopenia and cancer and the significant role of radio‐ and/or chemotherapy in the treatment of cancer, we think it is necessary to explore the relationship of them. First, our study enhances the understanding of sarcopenia, which, while commonly observed in the elderly, remains underexplored in cancer patients, particularly those undergoing radio‐ and/or chemotherapy. The incidence, impact, and mechanisms of sarcopenia in this group are not widely understood. Our systematic review and meta‐analysis can enhance medical practitioners' understanding of the association between sarcopenia and cancer treatment. Second, our study emphasizes the impact of sarcopenia on treatment outcomes: current research mainly focuses on the efficacy and side effects of radio‐ and/or chemotherapy, while the impact of patients' physical status, such as sarcopenia, on treatment outcomes is insufficiently explored. Identifying sarcopenia as a prognostic factor is of great significance for predicting the adverse effects of chemoradiotherapy and overall survival. Third, our research can provide guidance for clinical practice. Analysing the specific impact of sarcopenia on the adverse effects and overall survival of cancer patients received radio‐ and/or chemotherapy can help clinicians conduct a more comprehensive assessment of patients before treatment, thereby adjusting individualized treatment strategies, optimizing treatment plans, and improving patient outcomes, quality of life and survival. Fourth, our research can promote early intervention and comprehensive management. The identification of sarcopenia as an important prognostic factor in cancer treatment can promote early diagnosis and intervention of sarcopenia, including nutritional support and appropriate physical activity, to improve the patient's overall condition and reduce the adverse effects of radio‐ and/or chemotherapy, improving the therapeutic effect. Finally, the completion of this paper will provide basic data and theoretical basis for future related research, promote in‐depth research on the relationship between sarcopenia and the treatment of cancer patients, and promote the formulation and update of relevant clinical guidelines.

It is worth noting that in addition to the factors previously discussed, the stage of cancer and the duration of treatment are important variables that could affect the prognostic value of sarcopenia in cancer patients. Advanced stages of cancer are often associated with more aggressive disease progression and poorer overall prognosis, which could exacerbate the adverse effects of sarcopenia. Similarly, longer durations of treatment, especially those involving intensive therapies like chemotherapy and radiation, could lead to greater declines in muscle mass and function, thereby potentially amplifying the negative impact of sarcopenia. Regrettably, no related data were obtained from included studies. Future studies should aim to stratify analyses by cancer stage and treatment duration to better understand their interplay with sarcopenia and to provide more tailored prognostic information.

### 4.1 Strengths and Limitations

The current study has the following strengths. Firstly, in order to ensure the data quality and relevance of the meta‐analysis, this study established strict inclusion and exclusion criteria. Secondly, in order to assess the quality of included studies, standardized assessment tools such as the Newcastle‐Ottawa Scale were used for scoring. In this way, we ensured that the studies included in the analysis met certain scientific and methodological standards, enhancing the credibility and applicability of the study conclusions. Third, in view of the certain heterogeneity in the results, our study comprehensively used subgroup analysis, sensitivity analysis, meta‐regression analysis and other methods to explore the sources of heterogeneity. Through the above‐mentioned rigorous research design and analysis methods, the conclusions of this study are more rigorous and credible.

This study also has some limitations. Firstly, high heterogeneity was evident in the study, which was predictable and may be attributed in part to baseline characteristics of the population (gender, sex, tumour category etc.), treatment options (chemotherapy, radiotherapy or chemoradiotherapy), and differences in statistical methods (confounder adjustment). However, we believe that the impact of heterogeneity on the study results is limited, given the high consistency of most subgroup and sensitivity analyses with the main findings. Secondly, the results of this study are derived from observational cohort studies, potentially constrained by confounding factors such as patient gender and adjuvant treatment methods. However, all included studies provided data comparing sarcopenic and non‐sarcopenic populations, matched with several important covariates, indicating that patients with sarcopenia are at a consistently increased risk of adverse effects and mortality. Thirdly, almost all studies have assessed only muscle mass and not muscle strength or physical performance, making it difficult to fully analyse the impact of these factors on patient outcomes. In future studies, more attention should be paid to the comprehensive assessment of sarcopenia, including muscle strength and physical function, to provide more comprehensive evidence to support clinical decision‐making.

### 4.2 Implications

This meta‐analysis provided important clinical implications and direction for future research for the risk of adverse effects and mortality in patients with tumours and sarcopenia who received radio‐ and/or chemotherapy, and also provides statistics on the prevalence in the included studies. Considering that sarcopenia can easily affect the efficacy of radio‐ and/or chemotherapy, increase the adverse effects and reduce the overall survival of tumour patients with sarcopenia, and at the same time, radio‐ and/or chemotherapy are also major triggers for sarcopenia. Early screening and intervention of sarcopenia are of great significance to the overall prognosis of tumour patients. However, widespread attention has not been paid to sarcopenia; most of the treatments for patients with tumours in the current clinical environment are only targeted at cancer, while neglecting the treatment of sarcopenia. The best strategy for managing cancer combined with sarcopenia has not yet been established. Together, we suggested that (i) sarcopenia should be a part of the initial evaluation of tumour patients who received radio‐ and/or chemotherapy, (ii) patients with tumour, regardless of diagnosis, treatment, or tumour stage, should receive regular follow‐up for sarcopenia routinely, and (iii) additional studies are necessary to include sarcopenia into an outcome scale for tumour patients. The measure of integrating sarcopenia assessment into routine clinical practice could revolutionize cancer care by enabling personalized interventions to optimize treatment outcomes and enhance the patient's quality of life. Future research should prioritize the development of standardized methodologies for sarcopenia assessment, longitudinal studies to establish causality, and interventions targeting sarcopenia to mitigate toxicity associated with radiotherapy and/or chemotherapy. More efforts aimed at elucidating the mechanisms underlying the relationship between sarcopenia and toxic effects will facilitate the development of targeted interventions to minimize adverse effects and optimize treatment outcomes for patients battling malignant tumours.

## 5 Conclusion

In summary, this study demonstrated that sarcopenia had an impact on over one‐third of tumour patients who received radio‐ and/or chemotherapy and resulted in a higher risk of adverse effects and mortality. These findings elucidated the integral role of sarcopenia as a prognostic indicator for chemotherapy‐induced toxicity and mortality in patients with malignant tumours. Although the study strives to be rigorous in design and execution, there is still a certain degree of heterogeneity and publication bias due to factors such as the diversity of sarcopenia assessment methods, the complexity of tumour category, and differences in research backgrounds in different regions which prompt us to be cautious when interpreting the study results and to further explore these issues in future studies to improve the accuracy and applicability of the study.

## Author Contributions

Y.Z., J.Z. and Y.Z. are the co‐first authors. W.Y. and Q.L. designed the study. J.Z. and Y.Z. selected relevant articles. Z.P. interpreted data. Y.Z. was responsible for the writing of the report and literature search. All authors have read and approved the manuscript.

## Ethics Statement

Aggregate data were extracted from published studies; no patients were involved in this study; thus, ethical approval and informed consent were not required.

## Conflicts of Interest

The authors declare no conflicts of interest.

## Supporting information


**Table S1.** Detailed search strategy and process.

**Table S2.** Quality assessment of observational studies using Newcastle–Ottawa Scale.

**Table S3.** GRADE evidence profile: sarcopenia for adverse effects in patients with tumour received chemoradiotherapy.

**Table S4.** Sensitivity analysis for univariate analysis by excluding a study at a time and then pooling the remaining studies.

**Table S5.** Meta‐regression of toxic effects among sarcopenia vs. non‐sarcopenia groups.
**Table S6.** GRADE evidence profile: sarcopenia for mortality in patients with tumour received radio‐ and/or chemotherapy.
**Table S7.** Meta‐regression of overall survival among sarcopenia vs. non‐sarcopenia groups.

**Figure S1.** The pooled overall prevalence of tumour patients with sarcopenia in the included studies.
**Figure S2.** Subgroup analysis for prevalence of sarcopenia in patients with tumour received chemotherapy/chemoradiotherapy A. by different definitions of sarcopenia; B. by tumour category; C.by study location; D.by sex.
**Figure S3.** Sensitivity analysis of adverse effects.
**Figure S4.** A. Funnel plot of publication bias of adverse effects; B. Begg's funnel plot of publication bias of adverse effects; C. Eggers funnel plot of publication bias of adverse effects.
**Figure S5.** Sensitivity analysis of overall survival.
**Figure S6.** A. Funnel plot of publication bias of overall survival; B. Funnel plot of publication bias of overall survival; C. Eggers funnel plot of publication bias of overall survival.

## Data Availability

The datasets analysed during the current study will be available from the corresponding author upon reasonable request.

## Inclusion and Exclusion Criteria for Study Selection

Studies were included if they met the following criteria:

1. Participants: patients with malignant tumours confirmed by clinical/imaging or tissue biopsy criteria and received chemotherapy, radiotherapy or both of them.
2. Exposures: sarcopenia, defined by either muscle mass or muscle function parameters.
3. Outcomes: the association between sarcopenia, muscle mass, or muscle function and the risk of treatment‐related toxic effects, the risk of death and the prevalence of sarcopenia.
4. Study design: prospective or respective cohort study.

We excluded studies for the following reasons:

1. Studies with sample size less than 100.
2. Studies that not mentioned pre‐chemotherapy sarcopenia data.
3. Studies with insufficient data and no response from authors during our attempts to obtain additional relevant data and/or clarification of data.

## Quality Assessment of Observational Studies Using Newcastle–Ottawa Scale

In our quality assessment scale, studies were scored across three categories: selection (up to 4 points), comparability (up to 2 points) and outcome of study participants (up to 3 points). Studies with a cumulative score ≥ 7 were considered to be of high quality, 4 to 6 as moderate quality, and 0 to 3 as low quality.

*Note:* A study can be awarded a maximum of one star for each numbered item within the Selection and Outcome categories. A maximum of two stars can be given for Comparability Selection

1. Representativeness of the exposed cohort

a. truly representative of the average ______________ (describe) in the community*

b. somewhat representative of the average _____________ in the community*

c. selected group of users, e.g., nurses, volunteers

d. no description of the derivation of the cohort

2. Selection of the non‐exposed cohort

a. drawn from the same community as the exposed cohort*

b. drawn from a different source

c. no description of the derivation of the non‐exposed cohort

3. Ascertainment of exposure

a. secure record (e.g., surgical records)*

b. structured interview*

c. written self‐report

d. no description

4. Demonstration that outcome of interest was not present at start of study

a. yes*

b. no

Comparability:

1. Comparability of cohorts on the basis of the design or analysis.

a. study controls for _____________ (select the most important factor)*.

b. study controls for any additional factor* (This criterion could be modified to indicate specific control for a second important factor.)

Outcome:

1. Assessment of outcome

a. independent blind assessment*

b. record linkage*

c. self report

d. no description

2. Was follow‐up long enough for outcomes to occur

a. yes (select an adequate follow up period for outcome of interest)*

b. no

3. Adequacy of follow up of cohorts

a. complete follow up ‐ all subjects accounted for*

b. subjects lost to follow up unlikely to introduce bias—small number lost—> ____% (select an adequate %) follow up, or description provided of those lost)*

c. follow up rate < ____% (select an adequate %) and no description of those lost

d. no statement
