# Supplementary material for: Sarcopenia Is a Prognostic Factor of Adverse Effects and Mortality in Patients With Tumour: A Systematic Review and Meta‐Analysis
Source: J Cachexia Sarcopenia Muscle. 2024 Nov 11;15(6):2295–310. doi: 10.1002/jcsm.13629 (PMC11634529; doi:10.1002/jcsm.13629)
Supplement: Supplementary file 1 — Table S1. Detailed search strategy and process. [file JCSM-15-2295-s004.docx]

***Table S1.*** Detailed search strategy and process

| Search number | Query | Results |
| --- | --- | --- |
| PubMed search strategy | | |
| 1 | "Sarcopenia"[Mesh] | 11,249 |
| 2 | sarcopeni*[Title/Abstract] | 20,029 |
| 3 | myopeni*[Title/Abstract] | 96 |
| 4 | "muscle loss"[Title/Abstract] | 3,173 |
| 5 | "muscle depletion"[Title/Abstract] | 258 |
| 6 | "muscle wasting"[Title/Abstract] | 6,618 |
| 7 | "muscle reduction"[Title/Abstract] | 77 |
| 8 | "reduced muscle"[Title/Abstract] | 2,914 |
| 9 | "depleted muscle"[Title/Abstract] | 88 |
| 10 | "muscle attenuation"[Title/Abstract] | 332 |
| 11 | "muscle alteration"[Title/Abstract] | 51 |
| 12 | "loss of muscle"[Title/Abstract] | 4,782 |
| 13 | "muscle atrophy"[Title/Abstract] | 11,591 |
| 14 | "muscular atrophy"[Title/Abstract] | 11,100 |
| 15 | "Muscular Atrophy"[Mesh] | 24,042 |
| 16 | "muscle mass"[Title/Abstract] | 28,438 |
| 17 | "muscle index"[Title/Abstract] | 2,772 |
| 18 | "muscle thickness"[Title/Abstract] | 3,743 |
| 19 | "skeletal muscle"[Title/Abstract] | 128,364 |
| 20 | "Muscle, Skeletal"[Mesh] | 313,461 |
| 21 | "psoas muscle"[Title/Abstract] | 3,126 |
| 22 | "fat free mass"[Title/Abstract] | 10,074 |
| 23 | "lean mass"[Title/Abstract] | 7,999 |
| 24 | "body composition"[Title/Abstract] | 50,630 |
| 25 | "Body Composition"[Mesh] | 66,043 |
| 26 | "Malnutrition"[Mesh] | 138,994 |
| 27 | malnutrition[Title/Abstract] | 54,909 |
| 28 | "muscle function"[Title/Abstract] | 15,305 |
| 29 | "muscle strength"[Title/Abstract] | 32,964 |
| 30 | "muscular strength"[Title/Abstract] | 4,700 |
| 31 | "Muscle Strength"[Mesh] | 47,946 |
| 32 | "muscle power"[Title/Abstract] | 3,089 |
| 33 | "muscle fatigue"[Title/Abstract] | 5,895 |
| 34 | "Muscle Fatigue"[Mesh] | 9,500 |
| 35 | "muscle weakness"[Title/Abstract] | 19,586 |
| 36 | "muscular weakness"[Title/Abstract] | 2,190 |
| 37 | "Muscle Weakness"[Mesh] | 9,977 |
| 38 | "handgrip strength"[Title/Abstract] | 6,423 |
| 39 | "grip strength"[Title/Abstract] | 17,661 |
| 40 | "Hand Strength"[Title/Abstract] | 819 |
| 41 | "Hand Strength"[Mesh] | 22,030 |
| 42 | 1 OR 2 OR 3 OR 4 OR 5 OR 6 OR 7 OR 8 OR 9 OR 10 OR 11 OR  12 OR 13 OR 14 OR 15 OR 16 OR 17 OR 18 OR 19 OR 20 OR 21  OR 22 OR 23 OR 24 OR 25 OR 26 OR 27 OR 28 OR 29 OR 30 OR  31 OR 32 OR 33 OR 34 OR 35 OR 36 OR 37 OR 38 OR 39 OR 40  OR 41 OR 42 | 735,841 |
| 43 | "Neoplasms"[Mesh] | 4,010,093 |
| 44 | cancer[Title/Abstract] | 2,333,976 |
| 45 | tumor[Title/Abstract] | 1,511,978 |
| 46 | neoplasm[Title/Abstract] | 98,224 |
| 47 | malignancy[Title/Abstract] | 192,698 |
| 48 | carcinoma[Title/Abstract] | 747,582 |
| 49 | 43 OR 44 OR 45 OR 46 OR 47 OR 48 | 5,110,347 |
| 50 | "Radiotherapy"[Mesh] | 213,195 |
| 51 | radiotherapy[Title/Abstract] | 232,614 |
| 52 | Radiation Therapy[Title/Abstract] | 98,036 |
| 53 | "Chemotherapy, Adjuvant"[Mesh] | 47,678 |
| 54 | Chemotherapy[Title/Abstract] | 474,645 |
| 55 | "Chemoradiotherapy"[Mesh] | 21,117 |
| 56 | "Neoadjuvant Therapy"[Mesh] | 31,101 |
| 57 | Chemoradiotherapy[Title/Abstract] | 29,206 |
| 58 | chemoradiation[Title/Abstract] | 16,910 |
| 59 | radiochemotherapy[Title/Abstract] | 6,214 |
| 60 | 50 OR 51 OR 52 OR 53 OR 54 OR 55 OR 56 OR 57 OR 58 OR 59 | 807,003 |
| 61 | "toxicity" [Subheading] | 512,369 |
| 62 | toxicity[Title/Abstract] | 506,516 |
| 63 | dose-limiting toxicity[Title/Abstract] | 6,603 |
| 64 | Chemotherapy toxicity[Title/Abstract] | 811 |
| 65 | chemotoxicity[Title/Abstract] | 310 |
| 66 | Radiotherapy toxicity[Title/Abstract] | 181 |
| 67 | "Drug-Related Side Effects and Adverse Reactions"[Mesh] | 136,943 |
| 68 | adverse events[Title/Abstract] | 225,113 |
| 69 | adverse reactions[Title/Abstract] | 39,944 |
| 70 | side effects[Title/Abstract] | 291,327 |
| 71 | outcome*[Title/Abstract] | 2,586,091 |
| 72 | death*[Title/Abstract] | 1,103,562 |
| 73 | mortality[Title/Abstract] | 1,065,494 |
| 74 | "Mortality"[Mesh] | 431,298 |
| 75 | "mortality" [Subheading] | 632,315 |
| 76 | Surviv*[Title/Abstract] | 1,520,023 |
| 77 | "Survival"[Mesh] | 4,943 |
| 78 | "Survival Analysis"[Mesh] | 339,310 |
| 79 | "Survival Rate"[Mesh] | 192,159 |
| 80 | prognos*[Title/Abstract] | 878,295 |
| 81 | "Prognosis"[Mesh] | 2,001,606 |
| 82 | 61 OR 62 OR 63 OR 64 OR 65 OR 66 OR 67 OR 68 OR 69 OR 70 OR 71 OR 72 OR 73 OR 74 OR 75 OR 76 OR 77 OR 78 OR 79 OR 80 | 6,972,829 |
| 83 | regression[Title/Abstract] | 1,141,424 |
| 84 | "Regression Analysis"[Mesh] | 466,739 |
| 85 | prospective*[Title/Abstract] | 935,306 |
| 86 | "Longitudinal Studies"[Mesh] | 174,887 |
| 87 | "Prospective Studies"[Mesh] | 695,330 |
| 88 | clinical trial*[Title/Abstract] | 538,452 |
| 89 | "Clinical Trials as Topic"[Mesh] | 396,307 |
| 90 | observational[Title/Abstract] | 300,879 |
| 91 | "Observational Studies as Topic"[Mesh] | 9,990 |
| 92 | retrospective*[Title/Abstract] | 1,139,322 |
| 93 | "Retrospective Studies"[Mesh] | 1,224,576 |
| 94 | 83 OR 84 OR 85 OR 86 OR 87 OR 88 OR 89 OR 90 OR 91 OR 92 OR 93 | 4,450,850 |
| 95 | 42 AND 49 AND 60 AND 82 AND 94 | 2,192 |
| 96 | Review[Publication Type] | 3,377,767 |
| 97 | Editorial[Publication Type] | 701,713 |
| 98 | Letter[Publication Type] | 1,267,437 |
| 99 | Comment[Publication Type] | 1,040,823 |
| 100 | Case Reports[Publication Type] | 2,427,805 |
| 101 | 96 OR 97 OR 98 OR 99 OR 100 | 7,665,351 |
| 102 | 94 NOT 101 | 2,010 |
| 103 | 102 AND [Filters: Humans] | 1,759 |
| Embase search strategy | | |
| #116 | #114 AND #115 | 11,145 |
| #115 | 'human'/de | 27,868,770 |
| #114 | #99 NOT #113 | 11,371 |
| #113 | #100 OR #101 OR #102 OR #103 OR #104 OR #105 OR #106 OR #107 OR #108 OR #109 OR #110 OR #111 OR #112 | 9,846,724 |
| #112 | 'note'/exp | 932,816 |
| #111 | 'data paper'/exp | 75 |
| #110 | 'erratum'/exp | 300,399 |
| #109 | 'case report'/exp | 3,110,002 |
| #108 | 'editorial'/exp | 783,230 |
| #107 | 'letter'/exp | 1,255,388 |
| #106 | 'review'/exp | 3,371,550 |
| #105 | 'review':it | 3,295,818 |
| #104 | 'note':it | 983,610 |
| #103 | 'letter':it | 1,326,874 |
| #102 | 'erratum':it | 307,669 |
| #101 | 'editorial':it | 811,946 |
| #100 | 'data papers':it | 174 |
| #99 | #48 AND #59 AND #70 AND #87 AND #98 | 6,828,772 |
| #98 | #88 OR #89 OR #90 OR #91 OR #92 OR #93 OR #94 OR #95 OR #96 OR #97 | 6,828,772 |
| #97 | 'retrospective*':ab,ti | 1,875,982 |
| #96 | 'observational':ab,ti | 462,959 |
| #95 | 'clinical trial*':ab,ti | 753,716 |
| #94 | prospective*:ab,ti | 1,456,126 |
| #93 | regression:ab,ti | 1,607,593 |
| #92 | 'retrospective study'/exp | 1,666,705 |
| #91 | 'observational study'/exp | 388,703 |
| #90 | 'clinical trial'/exp | 1,946,183 |
| #89 | 'prospective study'/exp | 933,226 |
| #88 | 'regression analysis'/exp | 573,829 |
| #87 | #71 OR #72 OR #73 OR #74 OR #75 OR #76 OR #77 OR #78 OR #79 OR #80 OR #81 OR #82 OR #83 OR #84 OR #85 OR #86 | 9,667,607 |
| #86 | 'adverse events':ab,ti | 390,092 |
| #85 | toxicity:ab,ti | 685,231 |
| #84 | prognos*:ab,ti | 2,216,944 |
| #83 | surviv*:ab,ti | 2,216,944 |
| #82 | mortality:ab,ti | 1,563,960 |
| #81 | death*:ab,ti | 1,572,792 |
| #80 | outcome*:ab,ti | 3,766,796 |
| #79 | 'prognostic assessment'/exp | 31,858 |
| #78 | 'prognosis'/exp | 980,981 |
| #77 | 'survival analysis'/exp | 57,593 |
| #76 | 'survival'/exp | 1,523,126 |
| #75 | 'mortality risk'/exp | 53,539 |
| #74 | 'mortality'/exp | 1,492,517 |
| #73 | 'death'/exp | 2,180,714 |
| #72 | 'adverse events'/exp | 1,128,540 |
| #71 | 'toxicity'/exp | 826,631 |
| #70 | #60 OR #61 OR #62 OR #63 OR #64 OR #65 OR #66 OR #67 OR #68 OR #69 | 1,712,131 |
| #69 | 'radiochemotherapy':ab,ti | 9,352 |
| #68 | 'chemoradiation':ab,ti | 32,011 |
| #67 | 'chemoradiotherapy':ab,ti | 45,580 |
| #66 | 'chemotherapy':ab,ti | 756,154 |
| #65 | 'radiation therapy':ab,ti | 150,734 |
| #64 | radiotherapy:ab,ti | 339,182 |
| #63 | 'neoadjuvant therapy'/exp | 60,767 |
| #62 | 'chemoradiotherapy'/exp | 81,733 |
| #61 | 'chemotherapy'/exp | 877,862 |
| #60 | 'radiotherapy'/exp | 732,013 |
| #59 | #49 OR #50 OR #51 OR #52 OR #53 OR #54 OR #55 OR #56 OR #57 OR #58 | 7,279,737 |
| #58 | carcinoma:ab,ti | 999,636 |
| #57 | malignancy:ab,ti | 300,603 |
| #56 | tumor:ab,ti | 2,077,013 |
| #55 | cancer:ab,ti | 3,264,655 |
| #54 | neoplasms:ab,ti | 149,392 |
| #53 | 'carcinoma'/exp | 1,627,735 |
| #52 | 'malignancy'/exp | 25 |
| #51 | 'tumor'/exp | 6,398,399 |
| #50 | 'cancer'/exp | 4,800,870 |
| #49 | 'neoplasms'/exp | 6,398,399 |
| #48 | #1 OR #2 OR #3 OR #4 OR #5 OR #6 OR #7 OR #8 OR #9 OR #10 OR #11 OR #12 OR #13 OR #14 OR #15 OR #16 OR #17 OR #18 OR #19 OR #20 OR #21 OR #22 OR #23 OR #24 OR #25 OR #26 OR #27 OR #28 OR #29 OR #30 OR #31 OR #32 OR #33 OR #34 OR #35 OR #36 OR #37 OR #38 OR #39 OR #40 OR #41 OR #42 OR #43 OR #44 OR #45 OR #46 OR #47 | 1,446,152 |
| #47 | 'hand strength':ab,ti | 765 |
| #46 | 'grip strength':ab,ti | 24,676 |
| #45 | 'handgrip strength':ab,ti | 8.603 |
| #44 | 'muscular weakness':ab,ti | 3,468 |
| #43 | 'muscle weakness':ab,ti | 31,930 |
| #42 | 'muscle fatigue':ab,ti | 6,757 |
| #41 | 'muscle power':ab,ti | 3,887 |
| #40 | 'muscular strength':ab,ti | 5,894 |
| #39 | 'muscle strength':ab,ti | 44,559 |
| #38 | 'muscle function':ab,ti | 19,821 |
| #37 | 'malnutrition':ab,ti | 75,684 |
| #36 | 'body composition':ab,ti | 67,836 |
| #35 | 'lean mass':ab,ti | 12,232 |
| #34 | 'fat free mass':ab,ti | 13,331 |
| #33 | 'psoas muscle':ab,ti | 4,609 |
| #32 | 'skeletal muscle':ab,ti | 156,613 |
| #31 | 'muscle thickness':ab,ti | 4,522 |
| #30 | 'muscle size':ab,ti | 2,911 |
| #29 | 'muscle index':ab,ti | 4,464 |
| #28 | 'muscle mass':ab,ti | 40,327 |
| #27 | 'muscular atrophy':ab,ti | 14,811 |
| #26 | 'muscle atrophy':ab,ti | 15,277 |
| #25 | 'loss of muscle':ab,ti | 7,188 |
| #24 | 'muscle alteration':ab,ti | 81 |
| #23 | 'muscle attenuation':ab,ti | 506 |
| #22 | 'depleted muscle':ab,ti | 102 |
| #21 | 'reduced muscle':ab,ti | 4,176 |
| #20 | 'muscle reduction':ab,ti | 106 |
| #19 | 'muscle wasting':ab,ti | 9,293 |
| #18 | 'muscle depletion':ab,ti | 416 |
| #17 | 'muscle loss':ab,ti | 4,542 |
| #16 | myopeni*:ab,ti | 160 |
| #15 | sarcopeni*:ab,ti | 26,695 |
| #14 | 'hand strength'/exp | 45,472 |
| #13 | 'grip strength'/exp | 37,745 |
| #12 | 'muscle weakness'/exp | 478,184 |
| #11 | 'muscle fatigue'/exp | 15,239 |
| #10 | 'muscle strength'/exp | 99,430 |
| #9 | 'muscle function'/exp | 30,612 |
| #8 | 'malnutrition'/exp | 218,772 |
| #7 | 'body composition'/exp | 137,544 |
| #6 | 'fat free mass'/exp | 8,146 |
| #5 | 'skeletal muscle'/exp | 458,696 |
| #4 | 'psoas muscle'/exp | 6,972 |
| #3 | 'muscle thickness'/exp | 4,778 |
| #2 | 'muscle mass'/exp | 42,369 |
| #1 | 'muscle atrophy'/exp | 67,300 |
| Web of science search strategy | | |
| 1 | TS=(sarcopeni* OR myopeni* OR “muscle loss” OR “muscle  Depletion” OR “muscle wasting” OR “muscle reduction” OR  “reduced muscle” OR “depleted muscle” OR “muscle  attenuation” OR “muscle alteration” OR “loss of muscle” OR  “muscle atrophy” OR “muscular atrophy” OR “muscle mass” OR “muscle index” OR “muscle size” OR “muscle thickness” OR “skeletal muscle” OR “psoas muscle” OR “fat free mass” OR “lean mass” OR “body composition” OR “malnutrition” OR “muscle function” OR “mus-cle strength ”OR “muscular strength” OR “muscle power” OR “muscle fatigue” OR “muscle weakness” OR “muscular weakness” OR “handgrip strength” OR “grip strength” OR “Hand Strength”)  Databases= WOS, BCI, CSCD, DIIDW, INSPEC, KJD, MEDLINE,  RSCI, SCIELO Timespan=All years  Search language=Auto | 1,076,344 |
| 2 | TS=(neoplasms OR cancer OR tumor OR malignancy OR carcinoma)  Databases= WOS, BCI, CSCD, DIIDW, INSPEC, KJD, MEDLINE,  RSCI, SCIELO Timespan=All years  Search language=Auto | 10,963,673 |
| 3 | TS=(radiotherapy OR "radiation therapy" OR chemotherapy OR chemoradiotherapy OR chemoradiation OR radiochemotherapy OR “neoadjuvant therapy”)  Databases= WOS, BCI, CSCD, DIIDW, INSPEC, KJD, MEDLINE,  RSCI, SCIELO Timespan=All years  Search language=Auto | 2,890,955 |
| 4 | TS=(toxicity OR “adverse events” OR death* OR mortality OR “mortality risk” OR Surviv* OR prognos* OR outcome*)  Databases= WOS, BCI, CSCD, DIIDW, INSPEC, KJD, MEDLINE,  RSCI, SCIELO Timespan=All years  Search language=Auto | 13,501,890 |
| 5 | TS=(retrospective* OR observational OR “clinical trial*” OR prospective*) | 4,784,230 |
| 6 | #1 AND #2 AND #3 AND #4 AND #5 | 3,523 |
| 7 | #6 and Editorial Material or Case Report or Review Article or Awarded Grant or Meeting or Abstract or Unspecified or Data Set or Dissertation Thesis or Early Access or Letter or Correction or Book or Data Study or Reference Material or Bibliography or Report or Retracted Publication (Exclude – Document Types) | 2,932 |
| 8 | TS=(mouse OR rat OR mice)  Databases= WOS, BCI, CSCD, DIIDW, INSPEC, KJD, MEDLINE,  RSCI, SCIELO Timespan=All years  Search language=Auto | 8,037,458 |
| 9 | #7 not #8  Databases= WOS, BCI, CSCD, DIIDW, INSPEC, KJD, MEDLINE,  RSCI, SCIELO Timespan=All years  Search language=Auto | 2,875 |
