# Supplementary material for: Sarcopenia Is a Prognostic Factor of Adverse Effects and Mortality in Patients With Tumour: A Systematic Review and Meta‐Analysis
Source: J Cachexia Sarcopenia Muscle. 2024 Nov 11;15(6):2295–310. doi: 10.1002/jcsm.13629 (PMC11634529; doi:10.1002/jcsm.13629)
Supplement: Supplementary file 2 — Table S2. Quality assessment of observational studies using Newcastle–Ottawa Scale. [file JCSM-15-2295-s003.docx]

***Table S2.*** Quality assessment of observational studies using Newcastle–Ottawa Scale.

|  | Selection | | | | Comparability | Outcome | | | Quality |
| --- | --- | --- | --- | --- | --- | --- | --- | --- | --- |
| Author(year) | Representativeness of the exposed cohort (1 point) | Selection of the non exposed cohort (1 point) | Ascertainment of exposure (1 point) | Demonstration that outcome of interest was not present at start of study (1 point) | Comparability of cohorts on the basis of the design or analysis (2 point) | Assessment of outcome (1 point) | Was follow-up long enough for outcomes to occur (1 point) | Adequacy of follow up of cohorts (1 point) | (9 point) |
| Abe, 2022 | 1 | 1 | 1 | 1 | 2 | 1 | 1 | 1 | 9 |
| Ahern, 2023 | 1 | 1 | 1 | 1 | 2 | 1 | 1 | 1 | 9 |
| Bedrikovetski, 2023 | 1 | 1 | 1 | 1 | 2 | 1 | 1 | 1 | 9 |
| Bruno,2021 | 1 | 1 | 0 | 1 | 2 | 1 | 1 | 1 | 8 |
| Charette, 2019 | 1 | 1 | 1 | 1 | 2 | 1 | 1 | 1 | 9 |
| Cho, 2018 | 1 | 1 | 1 | 1 | 2 | 1 | 1 | 1 | 9 |
| Choi, 2018 | 1 | 1 | 1 | 1 | 2 | 1 | 1 | 1 | 9 |
| Chiloiro, 2024 | 1 | 1 | 1 | 1 | 2 | 1 | 1 | 1 | 9 |
| Daly,2018 | 1 | 1 | 1 | 1 | 2 | 1 | 1 | 1 | 9 |
| Ganju,2019 | 1 | 1 | 1 | 1 | 2 | 1 | 1 | 1 | 9 |
| Go, 2016 | 1 | 1 | 1 | 1 | 2 | 1 | 1 | 1 | 9 |
| Hua, 2020 | 1 | 1 | 1 | 1 | 2 | 1 | 1 | 1 | 9 |
| Huang, 2019 | 1 | 1 | 1 | 1 | 2 | 1 | 1 | 1 | 9 |
| Huang, 2020 | 1 | 1 | 1 | 1 | 2 | 1 | 1 | 1 | 9 |
| Huang, 2021 | 1 | 0 | 1 | 1 | 2 | 1 | 1 | 1 | 8 |
| Ishizaki, 2023 | 1 | 1 | 1 | 1 | 2 | 1 | 1 | 1 | 9 |
| Jin, 2022 | 1 | 1 | 1 | 1 | 2 | 1 | 1 | 1 | 9 |
| Jang, 2024 | 1 | 1 | 1 | 1 | 2 | 1 | 1 | 1 | 9 |
| Kasahara, 2024 | 1 | 1 | 1 | 1 | 2 | 1 | 1 | 1 | 9 |
| Lee, 2018 | 1 | 1 | 1 | 1 | 1 | 1 | 1 | 1 | 8 |
| Lee,2021 | 1 | 1 | 1 | 1 | 2 | 1 | 1 | 1 | 9 |
| Li, 2023 | 1 | 1 | 1 | 1 | 2 | 1 | 1 | 1 | 9 |
| Liu, 2024 | 1 | 0 | 1 | 1 | 2 | 1 | 1 | 1 | 8 |
| Ma, 2021 | 1 | 1 | 1 | 1 | 2 | 1 | 1 | 1 | 9 |
| Martin, 2020 | 1 | 1 | 1 | 1 | 2 | 1 | 1 | 1 | 9 |
| Martin, 2022 | 1 | 1 | 1 | 1 | 2 | 1 | 1 | 1 | 9 |
| McSweeney, 2023 | 1 | 1 | 1 | 1 | 2 | 1 | 1 | 1 | 9 |
| Nilsson, 2021 | 1 | 1 | 1 | 1 | 2 | 1 | 1 | 1 | 9 |
| Pielkenrood, 2020 | 1 | 1 | 1 | 1 | 2 | 1 | 1 | 1 | 9 |
| Qian, 2022 | 1 | 1 | 1 | 1 | 1 | 1 | 1 | 1 | 8 |
| Stangl-Kremser, 2020 | 1 | 1 | 1 | 1 | 2 | 1 | 1 | 1 | 9 |
| Takeda,2018 | 1 | 1 | 1 | 1 | 2 | 1 | 1 | 1 | 9 |
| Thureau, 2021 | 1 | 1 | 1 | 1 | 1 | 1 | 1 | 1 | 8 |
| Watanabe, 2023 | 1 | 1 | 1 | 1 | 2 | 1 | 1 | 1 | 9 |
| Willemsen, 2019 | 1 | 1 | 1 | 1 | 1 | 1 | 1 | 1 | 8 |
| Xu, 2021 | 1 | 1 | 1 | 1 | 2 | 1 | 1 | 1 | 9 |
| Yamahara, 2021 | 1 | 1 | 1 | 1 | 2 | 1 | 1 | 1 | 9 |
| Yu,2020 | 1 | 1 | 1 | 1 | 2 | 1 | 1 | 1 | 9 |
| Zhang, 2021 | 1 | 1 | 1 | 1 | 2 | 1 | 1 | 1 | 9 |
