# Supplementary material for: Sarcopenia Is a Prognostic Factor of Adverse Effects and Mortality in Patients With Tumour: A Systematic Review and Meta‐Analysis
Source: J Cachexia Sarcopenia Muscle. 2024 Nov 11;15(6):2295–310. doi: 10.1002/jcsm.13629 (PMC11634529; doi:10.1002/jcsm.13629)
Supplement: Supplementary file 3 — Table S3. GRADE evidence profile: sarcopenia for adverse effects in patients with tumour received chemoradiotherapy. [file JCSM-15-2295-s002.docx]

***Table S3***. GRADE evidence profile: sarcopenia for adverse effects in patients with tumor received radio- and/or chemotherapy.

| Quality assessment | | | | | | | | Summary of findings | | | Grading  of evidence |
| --- | --- | --- | --- | --- | --- | --- | --- | --- | --- | --- | --- |
| Study  design | Rating down factors | | | | | | | Study number | Number of  patients | RR (95% CI) | ⊕○○○  Low |
|  | Risk of bias | Inconsistency | | Indirectness | Imprecision | | Publication bias | 19 | 4903 | 1.44 (95% CI 1.21-1.71) |  |
| Cohort  studies (-2) | No serious risk of bias (-0) | No serious Inconsistency  (-0) | | No serious Indirectness (-0) | No serious Imprecision (-0) | | Suspected (-1) |  |  |  |  |
|  | Rating up factors | | | | | | |  |  |  |  |
|  | Large effect | | Dose response gradient | | | Direction of plausible confounding | |  |  |  |  |
|  | NO (+0) | | NO (+0) | | | NO (+0) | |  |  |  |  |

***Table S6***. GRADE evidence profile: sarcopenia for mortality in patients with tumor received radio- and/or chemotherapy.

| Quality assessment | | | | | | | | Summary of findings | | | Grading  of evidence |
| --- | --- | --- | --- | --- | --- | --- | --- | --- | --- | --- | --- |
| Study  design | Rating down factors | | | | | | | Study number | Number of  patients | HR (95% CI) | ⊕⊕○○  Low |
|  | Risk of bias | Inconsistency | | Indirectness | Imprecision | | Publication bias | 31 | 7588 | 1.66 (95% CI 1.40-1.96) |  |
| Cohort  studies (-2) | No serious risk of bias (-0) | No serious Inconsistency  (-0) | | No serious Indirectness (-0) | No serious Imprecision (-0) | | No serious  Publication  bias  (-0) |  |  |  |  |
|  | Rating up factors | | | | | | |  |  |  |  |
|  | Large effect | | Dose response gradient | | | Direction of plausible confounding | |  |  |  |  |
|  | NO (+0) | | NO (+0) | | | NO (+0) | |  |  |  |  |
