# Supplementary material for: Sarcopenia Is a Prognostic Factor of Adverse Effects and Mortality in Patients With Tumour: A Systematic Review and Meta‐Analysis
Source: J Cachexia Sarcopenia Muscle. 2024 Nov 11;15(6):2295–310. doi: 10.1002/jcsm.13629 (PMC11634529; doi:10.1002/jcsm.13629)
Supplement: Supplementary file 4 — Table S4. Sensitivity analysis for univariate analysis by excluding a study at a time and then pooling the remaining studies. [file JCSM-15-2295-s001.docx]

**Table S4.** Sensitivity analysis for univariate analysis by excluding a study at a time and then pooling the remaining studies.

| Study | RR | 95%Cl | |
| --- | --- | --- | --- |
| Omitting Abe, 2022 | 1.48 | 1.26 | 1.76 |
| Omitting Cho, 2018 | 1.48 | 1.25 | 1.77 |
| Omitting Martin, 2022 | 1.47 | 1.23 | 1.76 |
| Omitting Martin, 2020 | 1.37 | 1.17 | 1.59 |
| Omitting Thureau, 2021 | 1.48 | 1.24 | 1.77 |
