# Supplementary material for: Sarcopenia Is a Prognostic Factor of Adverse Effects and Mortality in Patients With Tumour: A Systematic Review and Meta‐Analysis
Source: J Cachexia Sarcopenia Muscle. 2024 Nov 11;15(6):2295–310. doi: 10.1002/jcsm.13629 (PMC11634529; doi:10.1002/jcsm.13629)
Supplement: Supplementary file 5 — Table S5. Meta‐regression of toxic effects among sarcopenia vs. non‐sarcopenia groups. Table S6. GRADE evidence profile: sarcopenia for mortality in patients with tumour received radio‐ and/or chemotherapy. Table S7. Meta‐regression of overall survival among sarcopenia vs. non‐sarcopenia groups. [file JCSM-15-2295-s006.docx]

***Table S5.*** Meta-regression of toxic effects among sarcopenia vs. non-sarcopenia groups.

| Variables | Coefficient | SE | t value | P value | CI-Lower | CI-Upper |
| --- | --- | --- | --- | --- | --- | --- |
| Sample size | 0.9994 | 0.0058 | -1.09 | 0.339 | 0.9978 | 1.0010 |
| Average age | 1.0460 | 0.0279 | 1.69 | 0.167 | 0.9714 | 1.1264 |
| Proportion of males | 1.3824 | 0.4089 | 1.09 | 0.335 | 0.6081 | 3.1427 |
| Study location | 1.0958 | 0.1149 | 0.87 | 0.432 | 0.8189 | 1.4662 |
| Tumor category | 1.3192 | 0.1443 | 2.53 | 0.065 | 0.9735 | 1.7875 |
| Treatment options | 1.1669 | 0.2672 | 0.67 | 0.537 | 0.6179 | 2.2038 |
| The method sarcopenia assessed | 1.4256 | 0.2084 | 2.43 | 0.072 | 0.9500 | 2.1393 |

***Table S7.*** Meta-regression of overall survival among sarcopenia vs. non-sarcopenia groups.

| Variables | Coefficient | SE | t value | P value | CI-Lower | CI-Upper |
| --- | --- | --- | --- | --- | --- | --- |
| Sample size | -0.0001 | 0.0057 | -0.02 | 0.984 | -0.0121 | 0.0119 |
| Average age | -0.0199 | 0.1375 | -0.14 | 0.887 | -0.3086 | 0.2689 |
| Proportion of males | 0.1851 | 3.5724 | 0.05 | 0.959 | -7.3203 | 7.6904 |
| Study location | -0.0392 | 0.6125 | -0.06 | 0.950 | -1.3262 | 1.2477 |
| Tumor category | 0.0384 | 0.7804 | 0.05 | 0.961 | -1.6012 | 1.6780 |
| Treatment options | 0.4133 | 1.3943 | 0.30 | 0.770 | -2.5161 | 3.3427 |
| The method  sarcopenia assessed | 0.3996 | 1.8426 | 0.22 | 0.831 | -3.4716 | 4.2708 |

SE = standard error; CI = confidence interval;
