# Supplementary material for: Sarcopenia Is a Prognostic Factor of Adverse Effects and Mortality in Patients With Tumour: A Systematic Review and Meta‐Analysis
Source: J Cachexia Sarcopenia Muscle. 2024 Nov 11;15(6):2295–310. doi: 10.1002/jcsm.13629 (PMC11634529; doi:10.1002/jcsm.13629)

**Figure. S1**. The pooled overall prevalence of tumor patients with sarcopenia in the included studies.

***
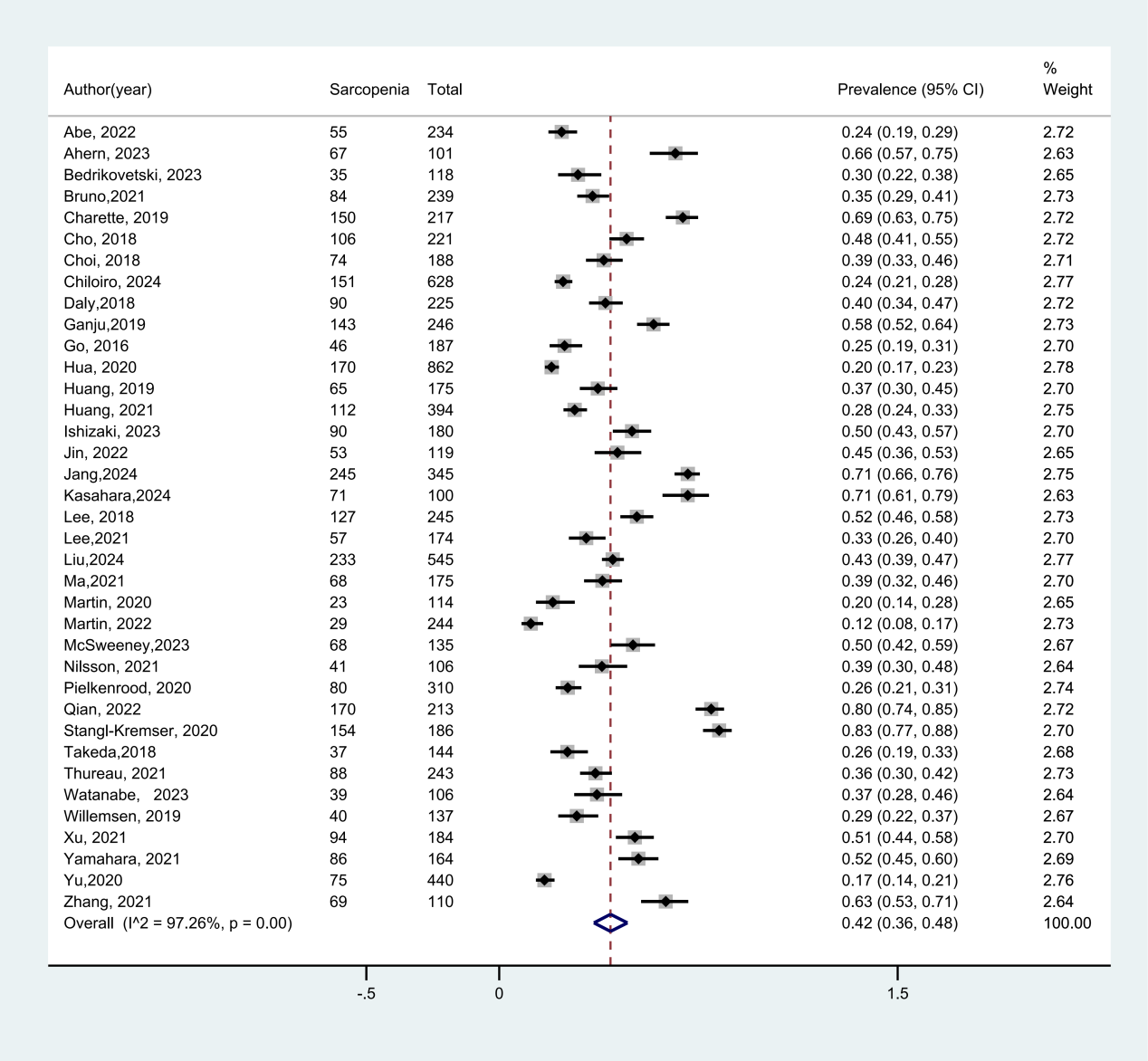
***

***Fig. S2A.*** Subgroup analysis for prevalence of sarcopenia in patients with tumor received chemotherapy/chemoradiotherapy by different definitions of sarcopenia


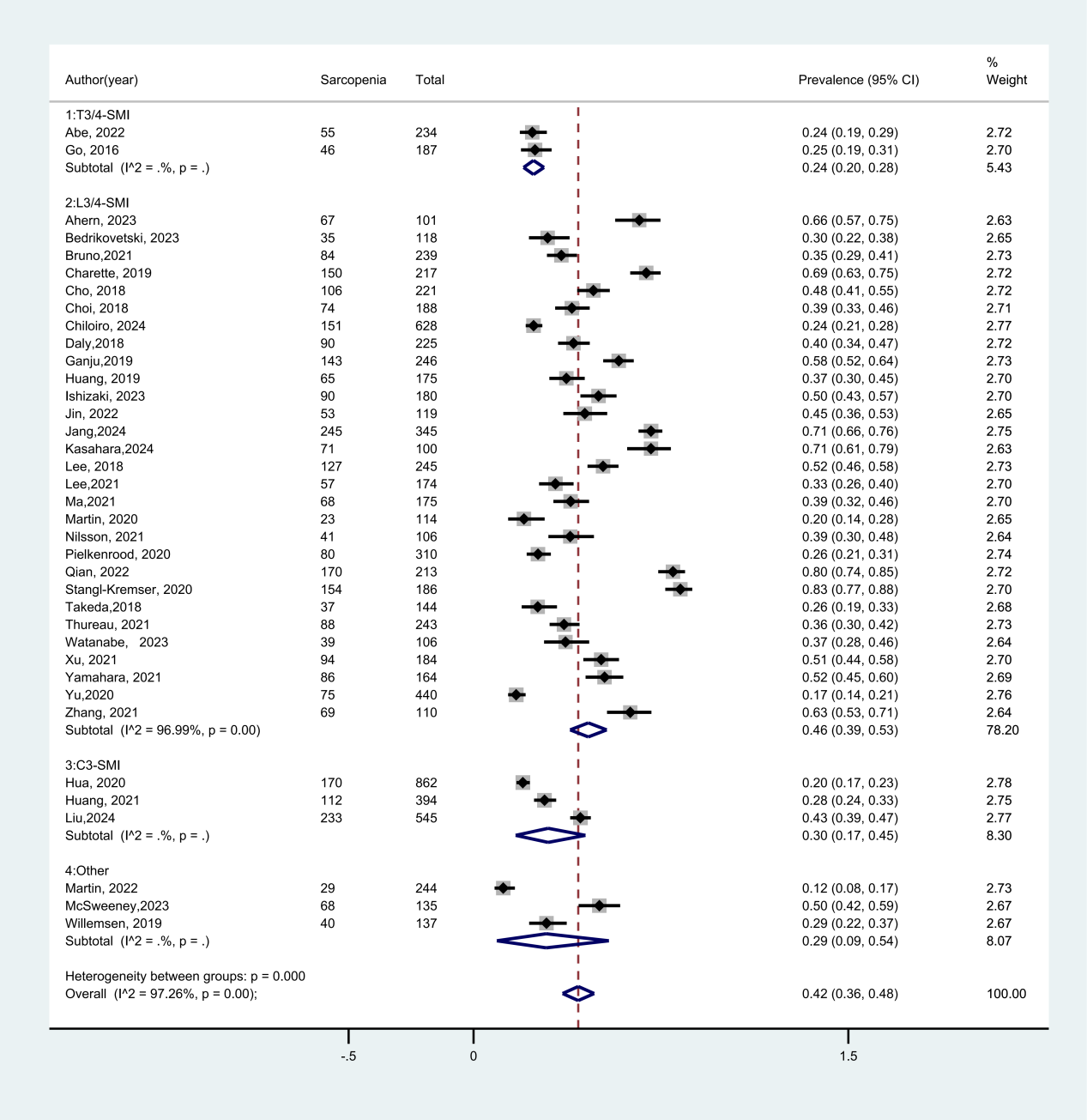


***Fig. S2B.*** Subgroup analysis for prevalence of sarcopenia in patients with tumor received radio- and/or chemotherapy by tumor category.


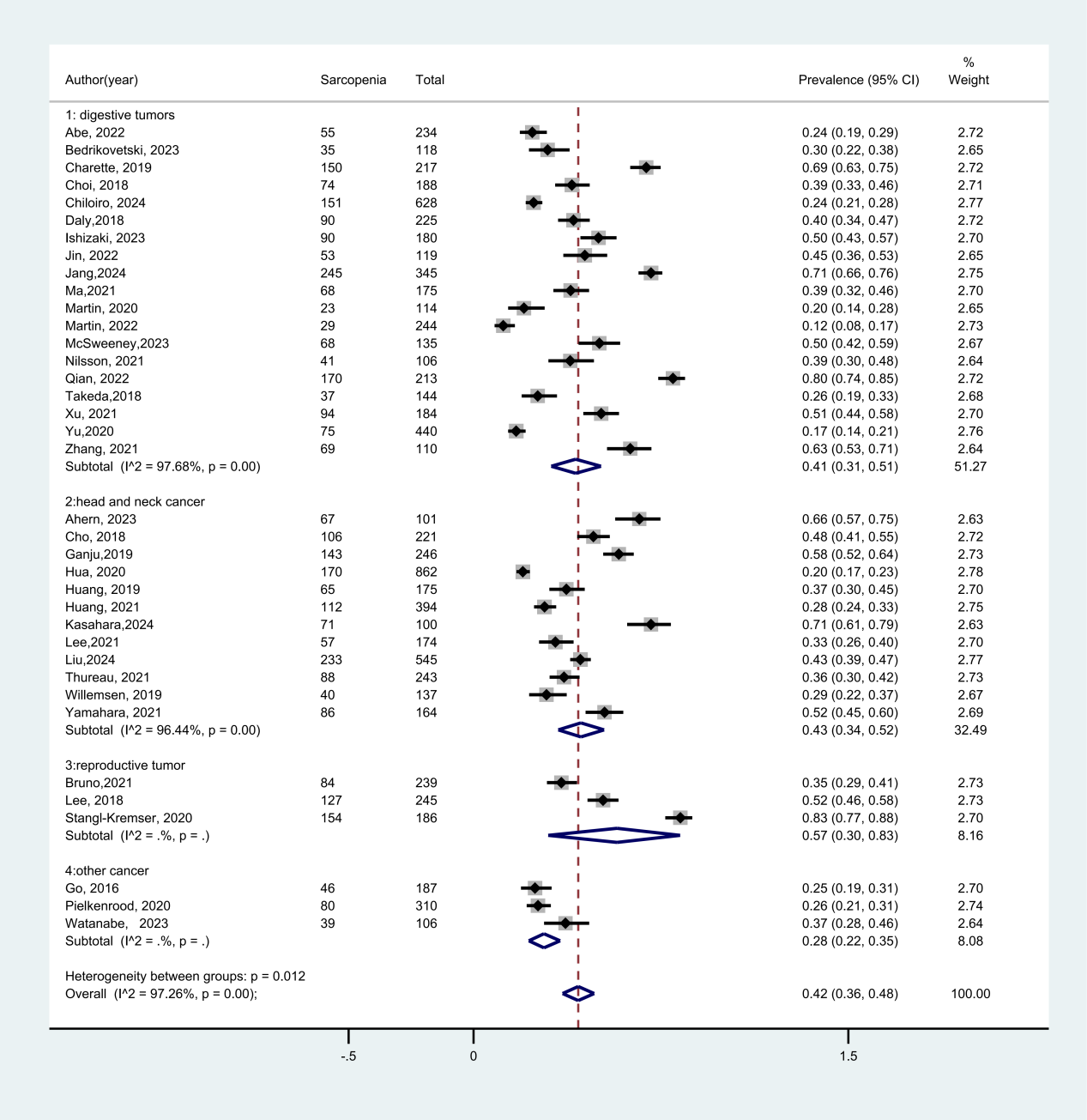


***Fig. S2C.*** Subgroup analysis for prevalence of sarcopenia in patients with tumors received radio- and/or chemotherapy by study location.

***Fig. S2D.*** Subgroup analysis for prevalence of sarcopenia in patients with tumor received radio- and/or chemotherapy by sex.

***Fig. S3.*** Sensitivity analysis of adverse effects.

***
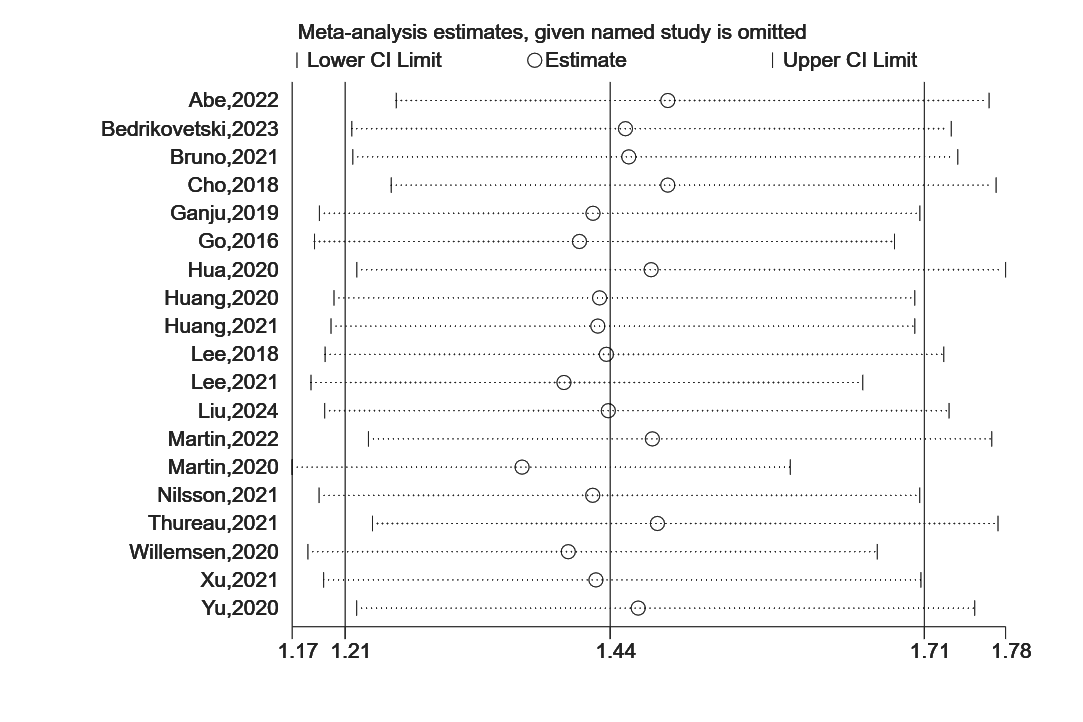
***

***Fig. S4A.*** Funnel plot of publication bias of adverse effects.

***
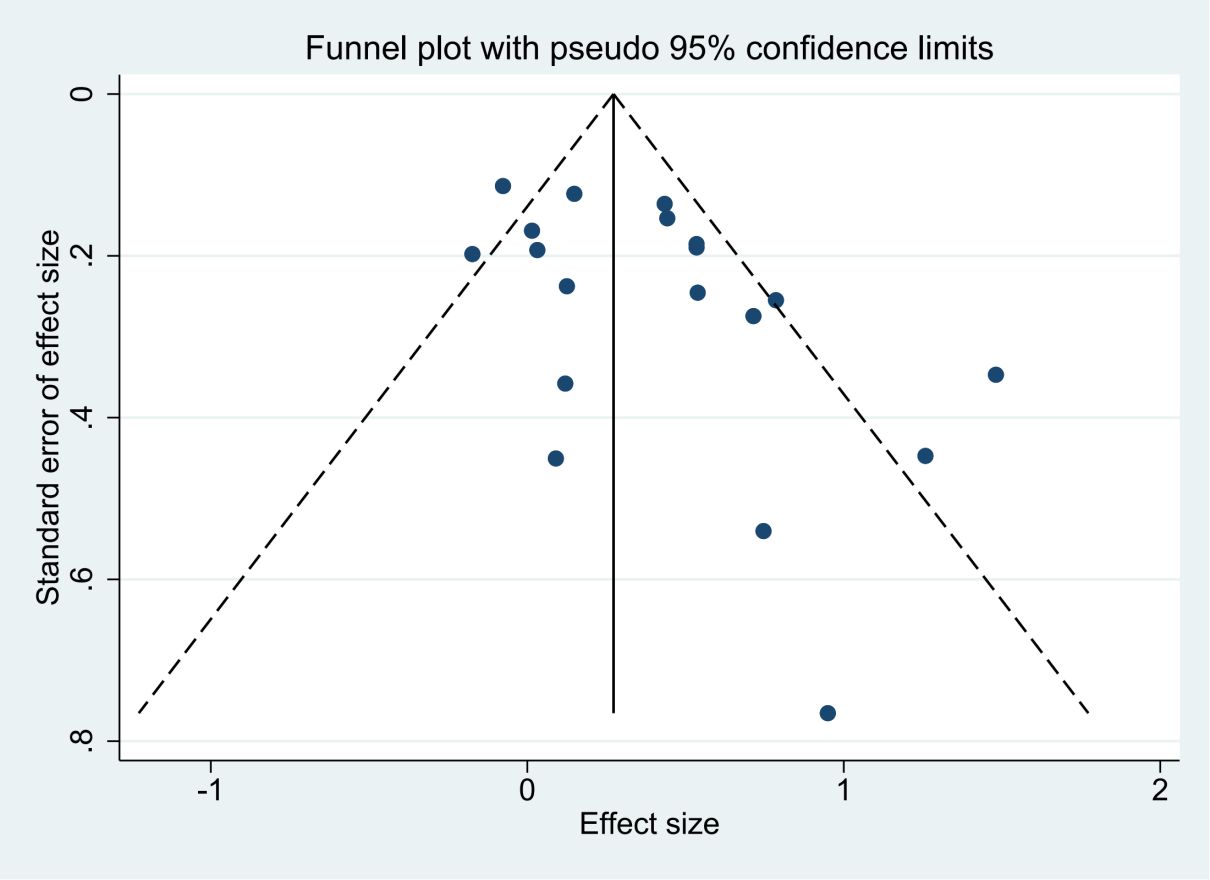
***

***Fig. S4B.*** Begg’s funnel plot of publication bias of adverse effects.

***
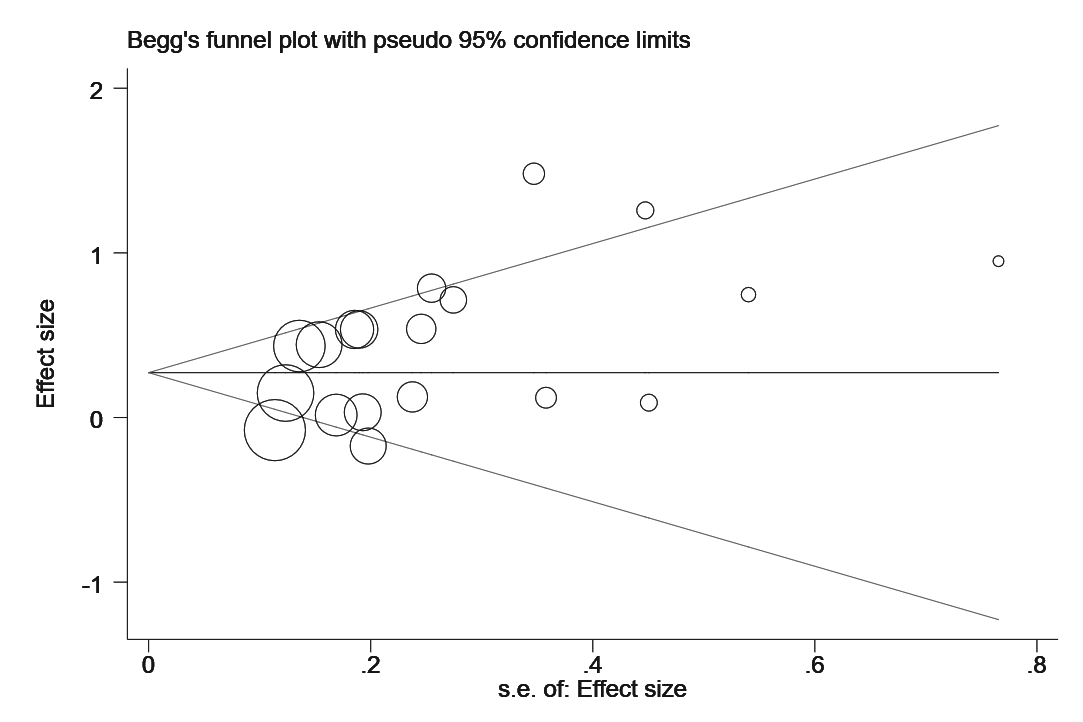
***

***Fig. S4C.*** Eggers funnel plot of publication bias of adverse effects.

*
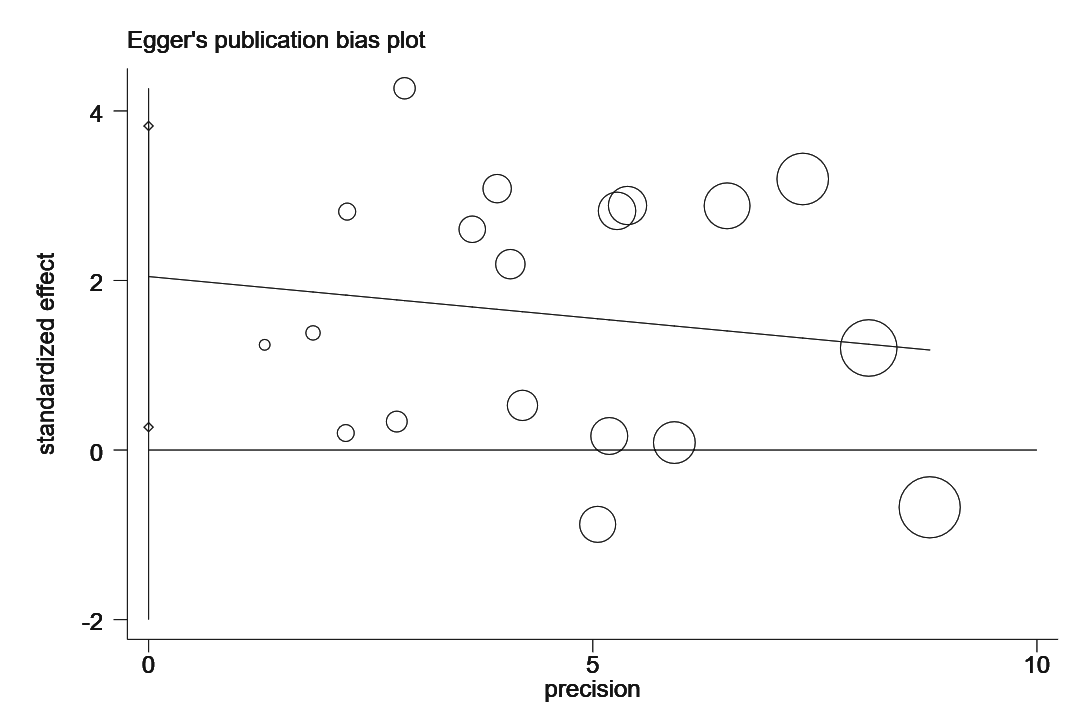
*

***Fig. S5.*** Sensitivity analysis of overall survival.


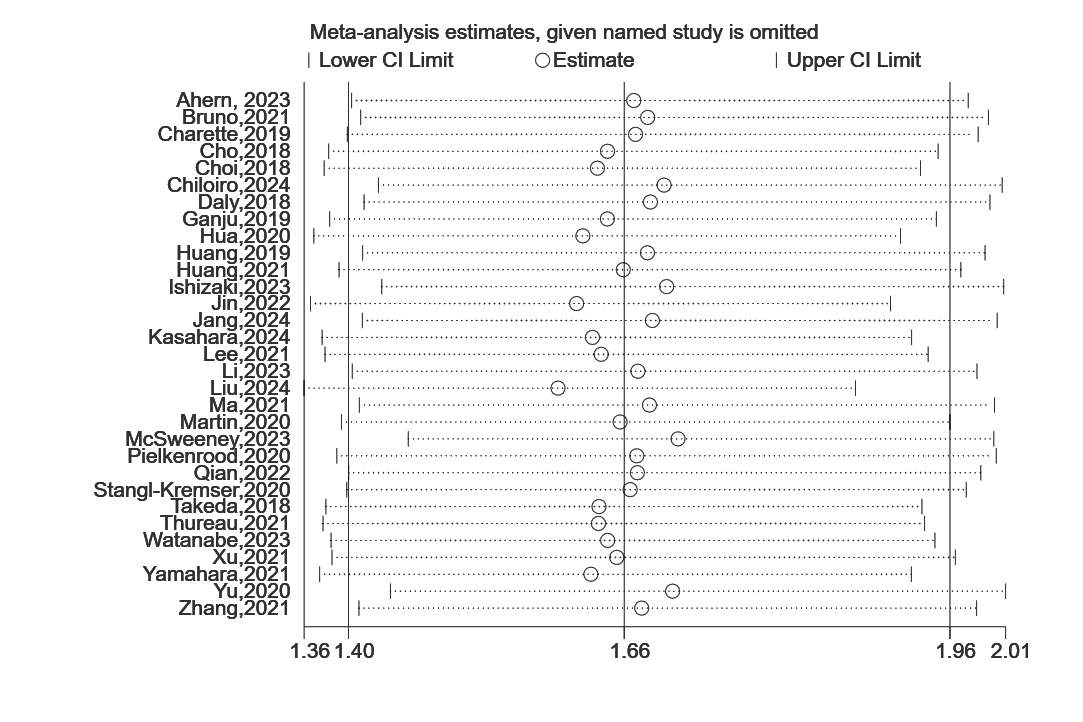


***Fig. S******6A.*** Funnel plot of publication bias of overall survival.


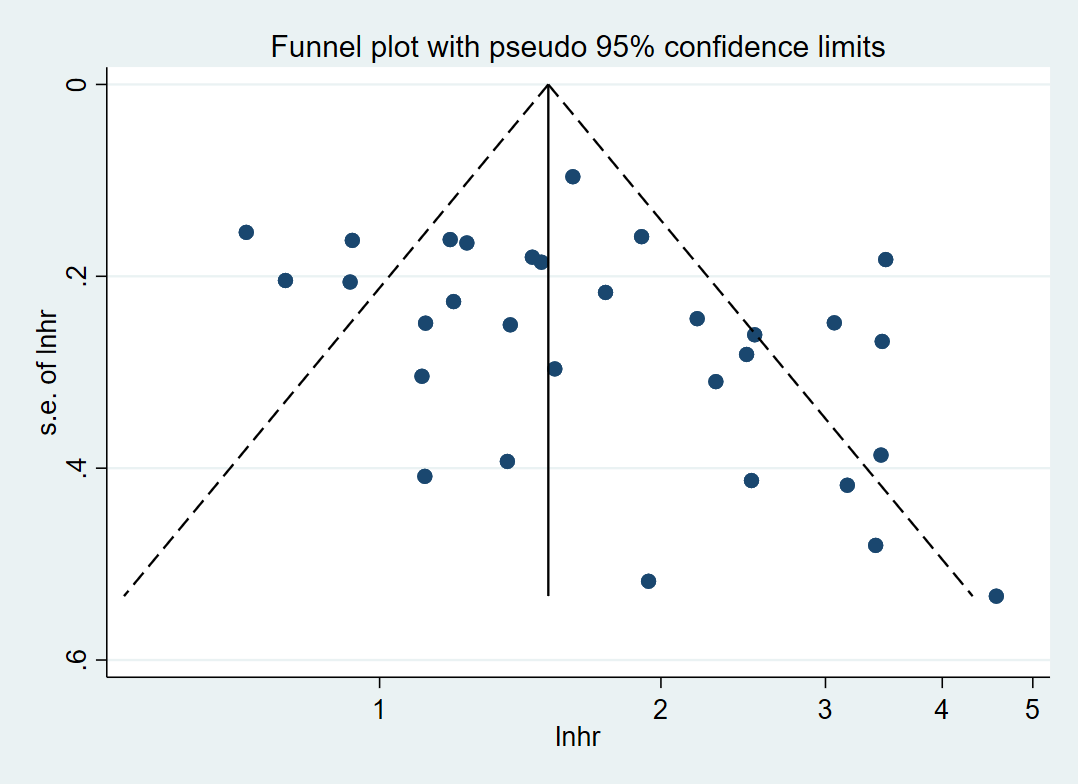


***Fig. S6B.*** Begg’s funnel plot of publication bias of overall survival.


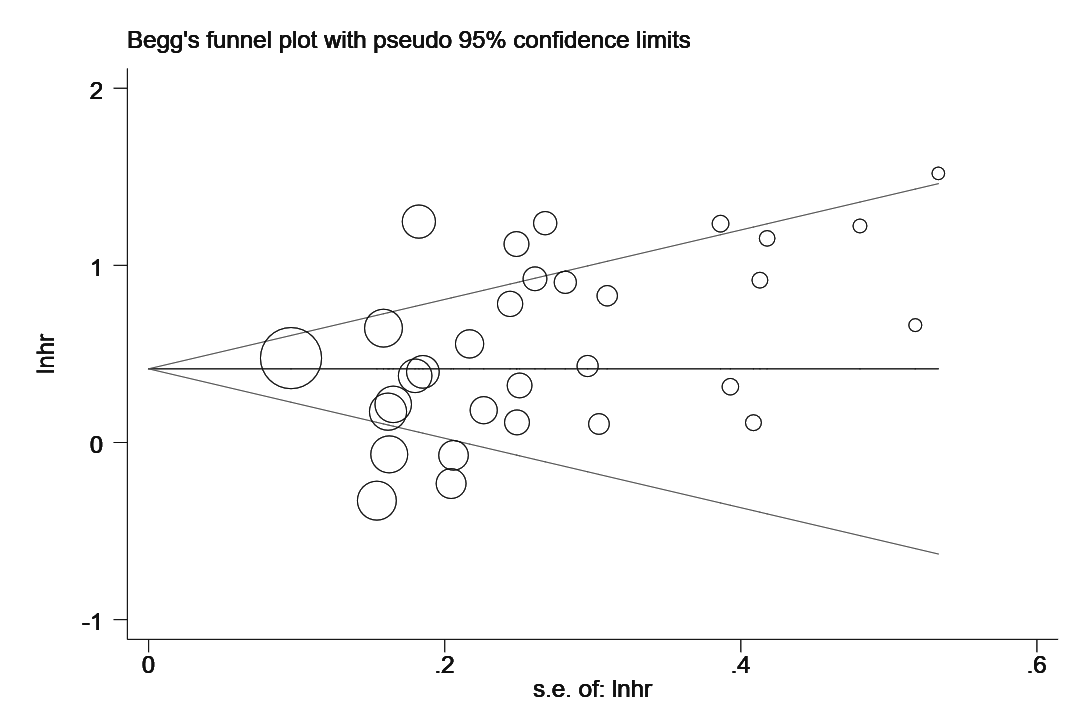


***Fig. S6C.*** Eggers funnel plot of publication bias of overall survival.


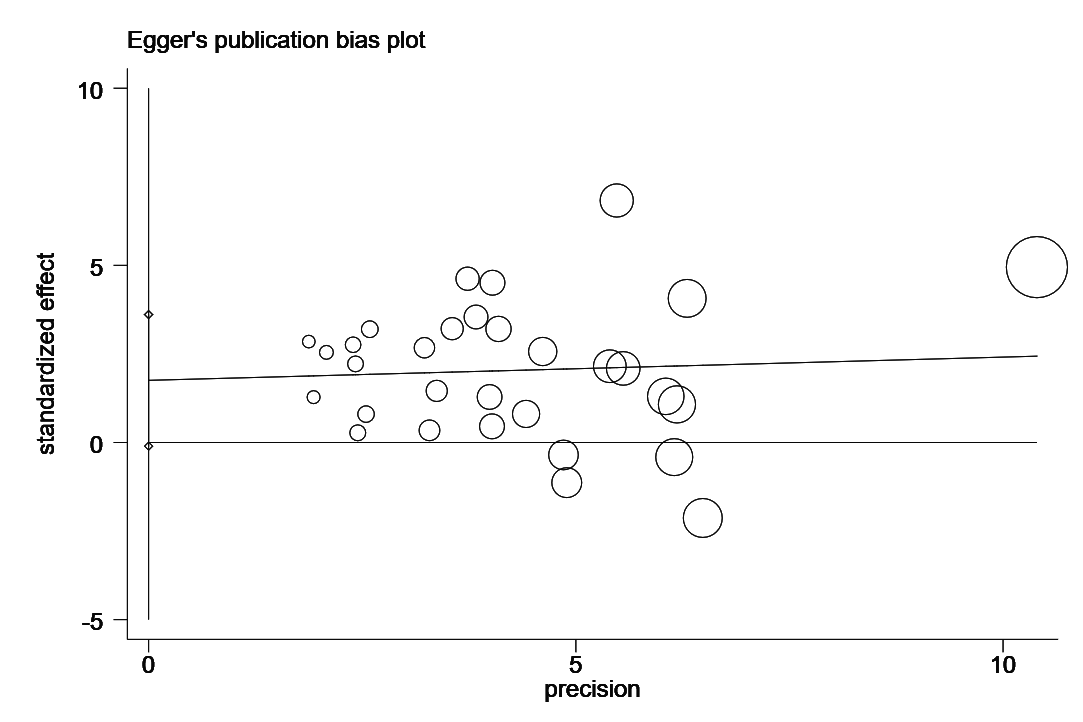

Supplement: Supplementary file 6 — Figure S1. The pooled overall prevalence of tumour patients with sarcopenia in the included studies. Figure S2. Subgroup analysis for prevalence of sarcopenia in patients with tumour received chemotherapy/chemoradiotherapy A. by different definitions of sarcopenia; B. by tumour category; C.by study location; D.by sex. Figure S3. Sensitivity analysis of adverse effects. Figure S4. A. Funnel plot of publication bias of adverse effects; B. Begg's funnel plot of publication bias of adverse effects; C. Eggers funnel plot of publication bias of adverse effects. Figure S5. Sensitivity analysis of overall survival. Figure S6. A. Funnel plot of publication bias of overall survival; B. Funnel plot of publication bias of overall survival; C. Eggers funnel plot of publication bias of overall survival. [file JCSM-15-2295-s005.docx]
